# Supplementary material for: Etiology and incidence of diarrhea requiring hospitalization in children under 5 years of age in 31 low- and middle-income countries: Findings from the Global Pediatric Diarrhea Surveillance network, 2017–2022
Source: PLOS Glob Public Health. 2026 Oct 27;6(10):e0006655. doi: 10.1371/journal.pgph.0006655 (PMC13712721; doi:10.1371/journal.pgph.0006655)
Supplement: Supplementary file 1 [file pgph.0006655.s001.pdf]

S1 Appendix. Global Pediatric Diarrhea Surveillance network Group Authors

Name	Affiliation
Fred Osei-Sarpong	World Health Organization, Accra, Ghana
Christabel Enweronu-Laryea	Korle-Bu Teaching Hospital, Accra, Ghana
Juanita Adams	Korle-Bu Teaching Hospital, Accra, Ghana
Makafui Seshie	Korle-Bu Teaching Hospital, Accra, Ghana
George Armah	Noguchi Memorial Institute for Medical Research, College of Health Sciences, University of Ghana, Accra, Ghana
Francis E. Dennis	Noguchi Memorial Institute for Medical Research, College of Health Sciences, University of Ghana, Accra, Ghana
Yashe Rimamdeyati	Nigeria Centre for Disease Control and Prevention, Abuja, Nigeria
Francis Ola	Nigeria Centre for Disease Control and Prevention, Abuja, Nigeria
Beckie Tagbo	Institute of Child Health, University of Nigeria Teaching Hospital, Enugu, Nigeria
Chinedu Chukwubike	Institute of Child Health, University of Nigeria Teaching Hospital, Enugu, Nigeria
Vina Okafor	Institute of Child Health, University of Nigeria Teaching Hospital, Enugu, Nigeria
Stella Okolie	Institute of Child Health, University of Nigeria Teaching Hospital, Enugu, Nigeria
Blessing Ekanem	World Health Organization, Abuja, Nigeria
Abdullahi Salihu Ahmed	World Health Organization, Abuja, Nigeria
Enya Bassey	World Health Organization, Abuja, Nigeria
Martin Chukwuji	World Health Organization, Abuja, Nigeria
Aristide Roch Sossou	World Health Organization, Cotonou, Benin
Annick Amoussou	Hôpital de Zone Suru Léré, Benin

J. M. Michel Agbla	Ministère de la Santé, Cotonou, Benin
Armel Hinvi	Ministère de la Santé, Cotonou, Benin
Britoh Mlan Alice	Yopougon University Hospital, Abidjan, Côte d'Ivoire
Boni Cisse	Yopougon University Hospital, Abidjan, Côte d'Ivoire
Zaba Flore Sandrine	Yopougon University Hospital, Abidjan, Côte d'Ivoire
Meite Syndou	Yopougon University Hospital, Abidjan, Côte d'Ivoire
Yapi Jaures	Yopougon University Hospital, Abidjan, Côte d'Ivoire
Ansah Julienne	Yopougon University Hospital, Abidjan, Côte d'Ivoire
Kouadio Sié Kabran	World Health Organization, Abidjan, Côte d'Ivoire
N'Zue Kofi	World Health Organization, Abidjan, Côte d'Ivoire
Koné Hamidou	Ministère de la Santé de l'Hygiène Publique et de la Couverture Maladie Universelle, Abidjan, Côte d'Ivoire
Gilles Tanguy M'Boutiki	World Health Organization, Ouagadougou, Burkina Faso
Joseph Nsiari Biey	World Health Organization, Ouagadougou, Burkina Faso
Ado Mpia Bwaka	World Health Organization, Ouagadougou, Burkina Faso
Pamela Mitula	World Health Organization, Ouagadougou, Burkina Faso
Mesfin Tefera	Ethiopian Public Health Institute, Addis Ababa, Ethiopia
Mekonnen Admassu	World Health Organization, Addis Ababa, Ethiopia

Julia Liliane Raboba	Tsaralalana Mother and Child Hospital, Antananarivo, Madagascar
Annick Lalaina Robinson	Tsaralalana Mother and Child Hospital, Antananarivo, Madagascar
Vonintsona Lalaina Rahajamanana	Tsaralalana Mother and Child Hospital, Antananarivo, Madagascar
Bahadoor Baboo Somduthsingh	Ministry of Health, Mauritius
David Mugisha	Mulago National Referral Hospital, Kampala, Uganda
Mulindwa Augustine	Mulago National Referral Hospital, Kampala, Uganda
Esther Nalumansi	Mulago National Referral Hospital, Kampala, Uganda
Hellen Tukamuhebwa Aanyu	Mulago National Referral Hospital, Kampala, Uganda
Cissy Nalunkuma	Uganda Martyrs Hospital – Lubaga, Kampala, Uganda
Elizabeth Kaudha	Naguru Referral Hospital, Kampala, Uganda
Annet Kisakye	World Health Organization, Kampala, Uganda
Mekess Kabwe	University Teaching Hospital, Lusaka, Zambia
Natasha Zimba	University Teaching Hospital, Lusaka, Zambia
Evans Mpabalwani	University Teaching Hospital, Lusaka, Zambia
Penelope Masumbu	World Health Organization, Lusaka, Zambia
Arnold Mukaratirwa	Parirenyatwa Group of Hospitals, Harare, Zimbabwe
Maxwell Rupfutse	World Health Organization, Harare, Zimbabwe
Goitom Weldegebriel	World Health Organization, Harare, Zimbabwe

Mapaseka L. Seheri	Diarrhoeal Pathogens Research Unit, Department of Virology, Sefako Makgatho Health Sciences University, Pretoria, South Africa
Nonkululeko Magagula	Diarrhoeal Pathogens Research Unit, Department of Virology, Sefako Makgatho Health Sciences University, Pretoria, South Africa
Kebareng Rakau-Nondela	Diarrhoeal Pathogens Research Unit, Department of Virology, Sefako Makgatho Health Sciences University, Pretoria, South Africa
Jeffrey Mphahlele	Diarrhoeal Pathogens Research Unit, Department of Virology, Sefako Makgatho Health Sciences University, Pretoria, South Africa
Archibald Worwui	World Health Organization Regional Office for Africa, Brazzaville, Congo
Jason M. Mwenda	World Health Organization Regional Office for Africa, Brazzaville, Congo
Julie Bines	Murdoch Children's Research Institute, Melbourne, Australia
Sarah Thomas	Murdoch Children's Research Institute, Melbourne, Australia
Hoa-Tran Nguyen	National Insitute for Hygiene and Epidemiology, Hanoi, Viet Nam
Reema Kumar	Fiji Centers for Disease Control, Suva, Fiji
Carren Bocaling	Research Institute for Tropical Medicine, Manila, Philippines
Joseph Bonifacio	Research Institute for Tropical Medicine, Manila, Philippines
Dandi Li	National Institute for Viral Disease Control and Prevention, China CDC, Beijing, China
Zhaojun Duan	Chinese Center for Disease Control and Prevention, Beijing, China

Lili Li	Chinese Center for Disease Control and Prevention, Beijing, China
Na Liu	Chinese Center for Disease Control and Prevention, Beijing, China
Varja Grabovac	Regional Office for the Western Pacific, World Health Organization, Manila, Philippines
Josephine Logronio	Regional Office for the Western Pacific, World Health Organization, Manila, Philippines
Batmunkh Nyambat	Regional Office for the Western Pacific, World Health Organization, Manila, Philippines
Tintu Varghese	The Wellcome Trust Research Laboratory, Division of Gastrointestinal Sciences, Christian Medical College, Vellore, India
Gagandeep Kang	The Wellcome Trust Research Laboratory, Division of Gastrointestinal Sciences, Christian Medical College, Vellore, India
Venkata Raghav Mohan	The Wellcome Trust Research Laboratory, Division of Gastrointestinal Sciences, Christian Medical College, Vellore, India
Sidhartha Giri	The Wellcome Trust Research Laboratory, Division of Gastrointestinal Sciences, Christian Medical College, Vellore, India
Dilip Abraham	The Wellcome Trust Research Laboratory, Division of Gastrointestinal Sciences, Christian Medical College, Vellore, India
Gagandeep Kang	The Wellcome Trust Research Laboratory, Division of Gastrointestinal Sciences, Christian Medical College, Vellore, India
Ira Praharaj	Indian Council of Medical Research Regional Medical Research Centre, Bhubaneswar, India
Ajithkumar V T	Calicut Medical College, Kerala, India
Fiji M D	Calicut Medical College, Kerala, India

Geeta Gathwala	Pandit Bhagwat Dayal Sharma Post Graduate Institute of Medical Sciences, Rohtak, India
Poonam Dalal	Pandit Bhagwat Dayal Sharma Post Graduate Institute of Medical Sciences, Rohtak, India
Manohar Badur	Sri Venkateshwara Medical College, Tirupati, India
Than Aye Nandar	World Health Organization, Yangon, Myanmar
Khaing Gyi	World Health Organization, Yangon, Myanmar
Htin Lin	Virology Research Division, Department of Medical Research, Yangon, Myanmar
Tondo Opute Emmanuel Njambe	World Health Organization Regional Office for South-East Asia, Delhi, India
Lucky Sangal	World Health Organization Regional Office for South-East Asia, Delhi, India
Seth Raman	World Health Organization Regional Office for South-East Asia, Delhi, India
Deepak Dhongde	World Health Organization Regional Office for South-East Asia, Delhi, India
Mohamed Sharifuzzaman	World Health Organization Regional Office for South-East Asia, Delhi, India
Svetlana Grigoryan	National Centre of Disease Control and Prevention, Yerevan, Armenia
Shushan Sargsyan	National Centre of Disease Control and Prevention, Yerevan, Armenia
Ion Birca	National Agency for Public Health, Ministry of Health, Chisinau, Republic of Moldova
Olga Burduniuc	National Agency for Public Health, Ministry of Health, Chisinau, Republic of Moldova
Makhtob Bobokholova	State Institution “Republican Center of Immunoprophylaxis”, Dushanbe, Tajikistan

Sudoba Suleymonova	State Institution “Republican Center of Immunoprophylaxis”, Dushanbe, Tajikistan
Farida Tishkova	Tajik Research Institute of Preventive Medicine, Dushanbe, Tajikistan
Manija Mullojonova	Tajik Research Institute of Preventive Medicine, Dushanbe, Tajikistan
Anvar Nazurdinov	World Health Organization, Dushanbe, Tajikistan
Liudmyla Chernyshova	National Medical Academy of Post-Graduate Education named after P.L. Shupyk, Kyiv, Ukraine
Natalya Radionova	Kiev City Children Clinical Hospital No.1, Kiev, Ukraine
Iryna Demchyshyna	Public Health Center of the Ministry of Health of Ukraine, Kyiv, Ukraine
Elena Samoilovich	Republican Research and Practical Center for Epidemiology and Microbiology, Ministry of Health, Minsk, Belarus
Galina Semeiko	Republican Research and Practical Center for Epidemiology and Microbiology, Ministry of Health, Minsk, Belarus
Roberta Pastore	World Health Organization Regional Office for Europe, Copenhagen, Denmark
Simarjit Singh	World Health Organization Regional Office for Europe, Copenhagen, Denmark
Dovile Videbaek	World Health Organization Regional Office for Europe, Copenhagen, Denmark
Danni S. Daniels	World Health Organization Regional Office for Europe, Copenhagen, Denmark
Tulio M. Fumian	Instituto Oswaldo Cruz Fiocruz, Rio de Janeiro, Brazil
Irene T.A. Maciel	Instituto Oswaldo Cruz Fiocruz, Rio de Janeiro, Brazil

Jose Paulo Gagliardi Leite	Instituto Oswaldo Cruz Fiocruz, Rio de Janeiro, Brazil
Lucia H. de Oliveira	Pan American Health Organization, Regional Office for the Americas, World Health Organization, Washington, DC, USA
Gloria Rey-Benito	Pan American Health Organization, Regional Office for the Americas, World Health Organization, Washington, DC, USA
Claudia Ortiz	Pan American Health Organization, Regional Office for the Americas, World Health Organization, Washington, DC, USA
Diana C. Caceres	Pan American Health Organization, Regional Office for the Americas, World Health Organization, Washington, DC, USA
Asad Ali	Department of Community Health Sciences, Aga Khan University, Karachi, Pakistan
Adil Kalam	Infectious Diseases Research Laboratory, Department of Paediatrics and Child Health, Aga Khan University, Karachi, Pakistan
Akram Hussain	Infectious Diseases Research Laboratory, Department of Paediatrics and Child Health, Aga Khan University, Karachi, Pakistan
Fatima Aziz	Infectious Diseases Research Laboratory, Department of Paediatrics and Child Health, Aga Khan University, Karachi, Pakistan
Furqan Kabir	Infectious Diseases Research Laboratory, Department of Paediatrics and Child Health, Aga Khan University, Karachi, Pakistan
Muhammad Adnan Kamran	Infectious Diseases Research Laboratory, Department of Paediatrics and Child Health, Aga Khan University, Karachi, Pakistan
Nimra Qadeer	Infectious Diseases Research Laboratory, Department of Paediatrics and Child Health, Aga Khan University, Karachi, Pakistan
Kamal Fahmy	Regional Office for the Eastern Mediterranean, World Health Organization, Cairo, Egypt

Amany Ghoniem	Regional Office for the Eastern Mediterranean, World Health Organization, Cairo, Egypt
Hmwe H. Kyu	Institute for Health Metrics and Evaluation, Seattle, Washington, USA
Matthew Doxey	Institute for Health Metrics and Evaluation, Seattle, Washington, USA
Elizabeth T Rogawski McQuade	Department of Epidemiology, Emory University, Atlanta, Georgia, USA
Matthew D. Esona	Centers for Disease Control and Prevention, Atlanta, Georgia, USA
Michael D. Bowen	Centers for Disease Control and Prevention, Atlanta, Georgia, USA
Gaston Bonenfant	Centers for Disease Control and Prevention, Atlanta, Georgia, USA
Rashi Gautam	Centers for Disease Control and Prevention, Atlanta, Georgia, USA
Sara A. Mirza	Centers for Disease Control and Prevention, Atlanta, Georgia, USA
Jan Vinjé	Centers for Disease Control and Prevention, Atlanta, Georgia, USA
Jacqueline E. Tate	Centers for Disease Control and Prevention, Atlanta, Georgia, USA
Umesh D. Parashar	Centers for Disease Control and Prevention, Atlanta, Georgia, USA
Jie Liu	Division of Infectious Diseases & International Health, University of Virginia, Charlottesville, VA, USA<br><br>School of Public Health, Qingdao University, Qingdao, Shandong, China
Darwin J. Operario	Division of Infectious Diseases & International Health, University of Virginia, Charlottesville, VA, USA

Timothy L. McMurry	Division of Infectious Diseases & International Health, University of Virginia, Charlottesville, VA, USA
Sarah E. Elwood	Division of Infectious Diseases & International Health, University of Virginia, Charlottesville, VA, USA
Eric R. Houpt	Division of Infectious Diseases & International Health, University of Virginia, Charlottesville, VA, USA
James A. Platts-Mills	Division of Infectious Diseases & International Health, University of Virginia, Charlottesville, VA, USA
Heidi M. Soeters	World Health Organization, Geneva, Switzerland
Sébastien Antoni	World Health Organization, Geneva, Switzerland
Shilpa S. Iyer	World Health Organization, Geneva, Switzerland
Mick N. Mulders	World Health Organization, Geneva, Switzerland
Tomoka Nakamura	World Health Organization, Geneva, Switzerland
Adam L. Cohen	World Health Organization, Geneva, Switzerland
Fatima Serhan	World Health Organization, Geneva, Switzerland
