# Supplementary material for: Etiology and incidence of diarrhea requiring hospitalization in children under 5 years of age in 31 low- and middle-income countries: Findings from the Global Pediatric Diarrhea Surveillance network, 2017–2022
Source: PLOS Glob Public Health. 2026 Oct 27;6(10):e0006655. doi: 10.1371/journal.pgph.0006655 (PMC13712721; doi:10.1371/journal.pgph.0006655)

S2 Appendix. Supplemental Material

Table of Contents

Table A. Pathogen-specific attributable fractions with 95% confidence intervals of diarrhea hospitalizations in children aged <5 years both overall and by geographic grouping in countries participating in Global Pediatric Diarrhea Surveillance, by two-year time periods during 2017-2022 (Underlying data for Figures 2 and 3). 2

Table B. Pathogen-specific attributable fractions with 95% confidence intervals of diarrhea hospitalizations in children aged <5 years both overall and by geographic grouping in countries participating in Global Pediatric Diarrhea Surveillance, limited to sites that had introduced rotavirus vaccine, by two-year time periods during 2017-2022 (Underlying data for Figure 4). 3

Table C. Pathogen-specific attributable fractions with 95% confidence intervals of diarrhea hospitalizations in children aged <5 years by Global Pediatric Diarrhea Surveillance site, 2021-2022 (Underlying data for Figure 5).... 4

Table D. Pathogen-specific attributable fractions with 95% confidence intervals of diarrhea hospitalizations in children aged <5 years by Global Pediatric Diarrhea Surveillance site, 2019-2020 (Underlying data for Figure 5).... 5

Table E. Pathogen-specific attributable fractions with 95% confidence intervals of diarrhea hospitalizations in children aged <5 years by Global Pediatric Diarrhea Surveillance site, 2017-2018 (Underlying data for Figure 5).... 6

Table F. All-cause and pathogen-specific attributable incidence per 1000 child-years with 95% confidence intervals of diarrhea hospitalizations in children aged <5 years both overall and by geographic grouping in countries participating in Global Pediatric Diarrhea Surveillance, by two-year time periods during 2017-2022 (Underlying data for Figure 6). 7

Fig A. Prevalence of pathogens including in the attribution modelling at cycle threshold cutoffs of 35 and 30 in children <5 years hospitalized with diarrhea and enrolled in Global Pediatric Diarrhea Surveillance, by site and two-year time period from 2017-2022. 8

Table A. Pathogen-specific attributable fractions with 95% confidence intervals of diarrhea hospitalizations in children aged <5 years both overall and by geographic grouping in countries participating in Global Pediatric Diarrhea Surveillance, by two-year time periods during 2017-2022 (Underlying data for Figures 2 and 3).

	**Rotavirus**	***Shigella***	**Norovirus**	**Adenovirus 40/41**	**Sapovirus**	**ETEC**	***Cryptosporidium***	**Astrovirus**	***C. jejuni/coli***	***Salmonella***	**tEPEC**	***V. cholerae***
**2017-2018**												
Overall	36.7 (28.7, 46.7)	10.6 (8.0, 13.6)	6.2 (4.7, 7.7)	6.9 (4.7, 9.0)	4.3 (2.7, 5.7)	4.8 (2.9, 6.3)	3.3 (2.3, 4.6)	3.1 (2.1, 4.2)	1.4 (1.0, 1.9)	1.4 (0.6, 2.2)	0.7 (0.3, 1.2)	0.6 (0.5, 0.7)
Central America	20.1 (14.6, 27.8)	17.3 (9.8, 25.9)	14.2 (7.2, 21.0)	9.2 (3.8, 14.3)	3.4 (1.5, 5.1)	2.6 (1.2, 4.1)	1.3 (0.6, 2.3)	5.1 (2.6, 7.6)	3.1 (0.3, 5.6)	0.5 (0.2, 0.8)	0.7 (0.0, 2.3)	0.0 (0.0, 0.0)
South America	13.0 (9.5, 17.2)	12.2 (8.9, 16.4)	23.7 (17.4, 31.3)	5.4 (3.5, 7.7)	4.2 (3.1, 5.6)	2.6 (1.8, 3.5)	0.6 (0.4, 0.8)	2.1 (1.4, 2.9)	2.8 (1.6, 4.1)	0.8 (0.2, 1.4)	0.2 (0.1, 0.4)	0.0 (0.0, 0.0)
Eastern Europe	33.2 (20.9, 51.1)	1.2 (0.9, 1.6)	8.0 (5.3, 11.9)	2.0 (1.2, 3.1)	2.9 (1.8, 4.5)	0.8 (0.5, 1.2)	0.1 (0.1, 0.2)	0.7 (0.4, 1.1)	4.4 (2.1, 7.5)	1.7 (0.4, 2.9)	0.3 (0.0, 0.9)	0.0 (0.0, 0.0)
Central and Western Asia	20.2 (14.1, 28.0)	10.5 (7.4, 14.3)	9.9 (7.1, 13.4)	3.4 (1.5, 5.3)	6.2 (4.4, 8.4)	3.8 (2.7, 5.3)	2.9 (1.6, 4.7)	3.2 (2.1, 4.7)	0.6 (0.3, 0.8)	0.1 (0.0, 0.2)	0.4 (0.1, 0.9)	0.0 (0.0, 0.0)
West Africa	47.9 (33.7, 66.7)	9.6 (5.4, 14.7)	3.9 (1.9, 5.7)	5.3 (2.1, 8.0)	4.4 (1.5, 6.7)	5.7 (2.4, 8.6)	3.4 (1.6, 5.7)	3.5 (1.7, 5.4)	0.4 (0.3, 0.5)	2.0 (0.5, 3.3)	0.4 (0.1, 0.8)	0.1 (0.1, 0.1)
East and Southern Africa	20.4 (16.8, 24.8)	14.3 (10.5, 19.0)	7.1 (4.7, 9.5)	4.7 (2.9, 6.5)	4.6 (3.4, 6.0)	6.3 (4.5, 8.4)	5.8 (4.1, 7.9)	2.7 (1.8, 3.9)	2.2 (0.9, 3.3)	0.6 (0.3, 0.8)	1.6 (0.5, 2.9)	0.7 (0.6, 0.9)
South Asia	23.4 (15.9, 34.8)	12.4 (5.2, 20.5)	10.1 (4.1, 16.7)	17.1 (7.5, 27.8)	5.3 (1.8, 8.9)	1.8 (0.9, 2.9)	1.4 (0.6, 2.7)	3.2 (1.4, 5.0)	3.4 (1.2, 5.7)	1.3 (0.5, 2.0)	1.0 (0.0, 3.0)	2.3 (1.5, 3.3)
Southeast Asia and Oceania	31.7 (24.7, 40.5)	6.1 (4.3, 8.6)	10.0 (7.7, 12.8)	5.6 (2.6, 8.1)	2.2 (1.4, 3.0)	1.3 (0.9, 1.8)	0.3 (0.2, 0.5)	2.4 (1.6, 3.4)	2.7 (1.4, 4.0)	0.4 (0.1, 0.7)	0.0 (0.0, 0.5)	0.5 (0.4, 0.7)
East Asia	26.9 (17.2, 40.3)	0.0 (0.0, 0.0)	12.4 (7.9, 18.5)	2.2 (1.2, 3.5)	1.3 (0.8, 1.9)	0.1 (0.0, 0.1)	0.0 (0.0, 0.0)	1.5 (0.9, 2.2)	0.7 (0.4, 1.1)	1.1 (0.5, 1.8)	0.1 (0.0, 0.3)	0.0 (0.0, 0.0)
**2019-2020**												
Overall	32.4 (23.1, 42.8)	8.7 (6.7, 10.7)	6.7 (4.7, 8.5)	11.9 (5.9, 17.5)	3.8 (2.1, 5.1)	3.3 (2.5, 4.0)	1.9 (1.4, 2.6)	1.7 (1.3, 2.1)	1.3 (0.9, 1.8)	0.6 (0.3, 0.9)	1.1 (0.4, 1.9)	0.7 (0.5, 1.0)
Central America	21.0 (15.1, 28.8)	12.0 (7.9, 17.1)	22.5 (12.8, 32.2)	8.7 (3.6, 13.9)	4.0 (2.0, 5.8)	1.7 (1.0, 2.5)	0.4 (0.1, 0.8)	3.7 (2.0, 5.7)	1.7 (0.7, 2.7)	0.2 (0.0, 0.3)	0.2 (0.0, 0.6)	0.0 (0.0, 0.0)
South America	13.0 (9.6, 17.4)	13.9 (9.9, 19.0)	19.4 (14.5, 25.4)	6.5 (4.1, 9.1)	5.1 (3.4, 7.2)	3.3 (2.3, 4.7)	0.4 (0.3, 0.5)	1.3 (0.8, 1.7)	1.9 (1.2, 2.8)	0.3 (0.1, 0.5)	0.2 (0.1, 0.4)	0.0 (0.0, 0.0)
Eastern Europe	23.5 (14.4, 36.6)	0.8 (0.5, 1.2)	12.1 (7.8, 18.5)	8.1 (3.4, 14.0)	1.7 (1.0, 2.6)	0.1 (0.1, 0.2)	0.0 (0.0, 0.1)	0.1 (0.0, 0.1)	6.3 (2.8, 11.0)	1.6 (0.5, 2.9)	0.3 (0.1, 0.6)	0.1 (0.1, 0.2)
Central and Western Asia	22.0 (13.6, 32.0)	13.3 (8.7, 19.5)	11.4 (7.3, 16.5)	6.5 (2.8, 10.2)	5.5 (3.1, 8.3)	0.8 (0.5, 1.2)	1.8 (0.7, 2.9)	2.6 (1.6, 4.1)	0.9 (0.4, 1.4)	0.2 (0.0, 0.4)	1.5 (0.0, 3.0)	0.0 (0.0, 0.0)
West Africa	45.3 (28.1, 64.9)	5.6 (2.9, 8.2)	6.2 (2.8, 8.9)	13.9 (2.9, 24.0)	3.8 (0.8, 5.8)	2.2 (1.2, 3.2)	1.5 (0.9, 2.6)	1.2 (0.6, 1.7)	0.8 (0.5, 1.2)	0.6 (0.2, 1.0)	0.9 (0.2, 2.1)	0.0 (0.0, 0.0)
East and Southern Africa	17.3 (14.2, 21.0)	15.4 (11.0, 20.4)	7.3 (4.3, 10.0)	6.8 (3.3, 9.6)	4.3 (2.9, 6.1)	5.0 (3.6, 6.8)	3.5 (2.3, 4.8)	3.1 (1.8, 4.6)	2.2 (0.7, 3.4)	0.1 (0.0, 0.1)	1.2 (0.3, 2.4)	0.0 (0.0, 0.0)
South Asia	13.0 (9.6, 17.6)	12.5 (7.2, 18.5)	6.3 (2.9, 10.0)	12.8 (7.5, 19.8)	4.1 (1.7, 6.4)	5.2 (3.0, 7.4)	1.9 (1.1, 2.9)	2.1 (1.2, 3.0)	2.0 (0.5, 3.8)	1.0 (0.4, 1.6)	1.6 (0.0, 4.0)	3.6 (2.5, 4.9)
Southeast Asia and Oceania	32.2 (23.5, 44.2)	2.9 (2.0, 4.5)	8.2 (5.6, 11.4)	9.6 (0.3, 15.3)	1.5 (0.7, 2.3)	0.8 (0.3, 1.5)	0.2 (0.1, 0.3)	0.6 (0.3, 1.0)	1.4 (0.4, 3.2)	0.9 (0.3, 1.5)	0.0 (0.0, 1.3)	0.0 (0.0, 0.0)
East Asia	19.9 (12.8, 30.1)	0.0 (0.0, 0.0)	13.3 (8.5, 20.3)	2.3 (1.1, 3.8)	1.3 (0.8, 1.9)	0.0 (0.0, 0.0)	0.1 (0.1, 0.2)	0.6 (0.4, 1.0)	0.7 (0.4, 1.3)	0.8 (0.2, 1.5)	0.1 (0.0, 0.3)	0.0 (0.0, 0.0)
**2021-2022**												
Overall	26.7 (20.7, 34.1)	10.1 (7.7, 12.6)	7.3 (5.0, 9.2)	7.0 (4.1, 9.7)	2.5 (1.7, 3.2)	2.4 (1.9, 3.0)	2.0 (1.3, 2.8)	1.2 (0.9, 1.6)	1.5 (1.0, 2.0)	1.0 (0.6, 1.3)	0.7 (0.3, 1.1)	1.4 (1.1, 1.8)
Central America	11.7 (8.8, 15.6)	9.5 (6.4, 13.2)	15.8 (7.4, 23.4)	6.1 (2.8, 9.3)	4.4 (2.2, 6.3)	2.7 (1.6, 3.9)	0.1 (0.0, 0.2)	2.2 (1.5, 3.2)	2.8 (1.1, 4.5)	1.2 (0.1, 2.1)	0.5 (0.0, 1.3)	0.0 (0.0, 0.0)
South America	8.7 (6.7, 11.3)	12.1 (9.0, 16.2)	21.4 (15.6, 28.7)	6.1 (4.1, 8.5)	3.2 (2.2, 4.3)	3.1 (2.2, 4.2)	0.2 (0.1, 0.3)	2.1 (1.4, 3.0)	3.6 (2.2, 5.1)	0.3 (0.1, 0.4)	0.1 (0.0, 0.2)	0.0 (0.0, 0.0)
Eastern Europe	23.7 (15.8, 35.2)	1.1 (0.8, 1.7)	12.3 (8.0, 18.1)	6.3 (3.4, 10.0)	0.6 (0.4, 1.0)	0.7 (0.5, 1.1)	0.1 (0.1, 0.2)	1.1 (0.6, 1.8)	3.0 (1.2, 5.1)	0.3 (0.0, 0.5)	0.3 (0.0, 0.6)	0.0 (0.0, 0.0)
Central and Western Asia	16.7 (10.9, 24.1)	9.6 (6.3, 14.0)	12.2 (7.5, 17.8)	4.1 (1.6, 6.3)	4.0 (2.6, 5.9)	1.3 (0.8, 1.9)	2.3 (1.1, 3.9)	0.4 (0.3, 0.6)	0.7 (0.3, 1.1)	0.0 (0.0, 0.1)	0.5 (0.0, 1.3)	0.0 (0.0, 0.0)
West Africa	36.0 (23.8, 50.7)	4.7 (2.5, 6.9)	7.1 (3.1, 10.5)	9.3 (3.8, 14.5)	2.1 (0.7, 3.3)	1.4 (0.8, 2.1)	1.9 (0.8, 3.5)	1.2 (0.5, 1.8)	0.3 (0.2, 0.4)	0.3 (0.1, 0.4)	0.5 (0.1, 1.2)	0.1 (0.1, 0.1)
East and Southern Africa	19.9 (16.5, 24.0)	18.4 (13.1, 24.8)	5.6 (3.6, 7.5)	5.1 (3.0, 7.2)	3.4 (2.2, 4.7)	5.2 (3.8, 6.8)	3.9 (2.6, 5.4)	1.4 (0.9, 1.9)	2.6 (1.4, 3.8)	1.2 (0.2, 2.1)	1.3 (0.4, 2.3)	0.0 (0.0, 0.0)
South Asia	15.0 (10.8, 20.9)	16.8 (7.8, 25.5)	5.7 (2.5, 9.4)	4.8 (2.6, 7.4)	2.6 (0.7, 4.2)	2.5 (1.2, 3.6)	1.0 (0.5, 1.7)	1.1 (0.5, 1.7)	2.3 (0.6, 4.0)	2.0 (0.6, 3.1)	0.6 (0.0, 1.5)	3.7 (2.6, 5.4)
Southeast Asia and Oceania	20.5 (14.7, 28.1)	5.4 (3.2, 8.5)	16.4 (10.3, 22.6)	2.9 (0.9, 4.5)	3.1 (1.7, 4.6)	1.3 (0.7, 2.0)	0.1 (0.1, 0.2)	1.7 (1.1, 2.5)	4.4 (2.0, 7.3)	2.3 (1.0, 3.5)	0.5 (0.0, 1.1)	7.5 (4.9, 11.1)
East Asia	15.0 (9.3, 23.0)	0.0 (0.0, 0.0)	9.4 (5.9, 14.7)	5.4 (2.8, 8.9)	0.5 (0.3, 0.8)	0.0 (0.0, 0.0)	0.1 (0.0, 0.1)	0.8 (0.5, 1.4)	0.4 (0.1, 0.7)	2.5 (0.6, 4.5)	0.1 (0.0, 0.3)	0.0 (0.0, 0.0)

Attributable fractions are expressed as a percent. tEPEC=typical enteropathogenic *E. coli*. ETEC=heat-stable enterotoxigenic *E. coli*

Table B. Pathogen-specific attributable fractions with 95% confidence intervals of diarrhea hospitalizations in children aged <5 years both overall and by geographic grouping in countries participating in Global Pediatric Diarrhea Surveillance, limited to sites that had introduced rotavirus vaccine, by two-year time periods during 2017-2022 (Underlying data for Figure 4).

	Rotavirus	*Shigella*	Norovirus	Adenovirus 40/41	Sapovirus	ETEC	*Cryptosporidium*	Astrovirus	*C. jejuni/coli*	*Salmonella*	tEPEC	*V. cholerae*
**2017-2018**												
Overall	19.2 (16.2, 22.5)	14.6 (11.2, 18.6)	7.2 (5.1, 9.2)	7.2 (4.7, 9.9)	4.3 (3.2, 5.6)	4.9 (3.6, 6.5)	4.2 (2.9, 5.7)	2.8 (2.0, 3.8)	2.3 (1.3, 3.2)	0.8 (0.5, 1.1)	1.5 (0.5, 2.6)	1.0 (0.7, 1.3)
Central America	20.1 (14.4, 27.3)	17.3 (10.2, 25.7)	14.1 (7.1, 21.1)	9.3 (4.1, 14.5)	3.4 (1.5, 5.1)	2.6 (1.2, 4.1)	1.3 (0.6, 2.3)	5.0 (2.5, 7.5)	3.2 (0.3, 5.6)	0.5 (0.2, 0.8)	0.7 (0.0, 2.3)	0.0 (0.0, 0.0)
South America	13.0 (9.6, 17.4)	12.2 (8.9, 16.2)	23.8 (17.5, 31.5)	5.4 (3.5, 7.7)	4.2 (3.1, 5.6)	2.6 (1.8, 3.4)	0.6 (0.4, 0.8)	2.1 (1.4, 2.9)	2.8 (1.6, 4.1)	0.8 (0.3, 1.3)	0.2 (0.1, 0.4)	0.0 (0.0, 0.0)
Eastern Europe	18.7 (12.3, 27.4)	6.7 (4.5, 9.6)	9.5 (6.3, 14.0)	6.9 (3.0, 10.8)	2.9 (1.8, 4.4)	1.3 (0.8, 2.0)	1.0 (0.5, 1.6)	0.5 (0.2, 0.7)	4.2 (1.8, 7.4)	2.3 (0.5, 3.8)	0.1 (0.0, 0.2)	0.0 (0.0, 0.0)
Central and Western Asia	20.3 (14.2, 28.0)	10.5 (7.4, 14.3)	10.0 (7.1, 13.4)	3.3 (1.5, 5.2)	6.2 (4.4, 8.5)	3.8 (2.7, 5.2)	2.9 (1.6, 4.6)	3.2 (2.1, 4.7)	0.6 (0.3, 0.8)	0.1 (0.0, 0.2)	0.4 (0.1, 0.8)	0.0 (0.0, 0.0)
West Africa	19.9 (14.5, 26.1)	9.4 (6.5, 12.7)	3.4 (2.4, 4.6)	3.8 (1.6, 6.2)	2.7 (2.0, 3.7)	2.9 (2.0, 4.1)	3.3 (1.9, 4.8)	0.7 (0.5, 1.0)	1.8 (0.7, 2.9)	0.9 (0.3, 1.8)	2.1 (0.6, 3.9)	0.0 (0.0, 0.0)
East and Southern Africa	19.2 (15.3, 24.0)	16.1 (11.4, 21.9)	7.7 (4.8, 10.4)	4.6 (2.7, 6.4)	4.4 (3.0, 6.0)	6.4 (4.3, 8.9)	5.2 (3.3, 7.5)	2.9 (1.8, 4.4)	2.5 (0.9, 3.9)	0.6 (0.3, 0.9)	1.4 (0.1, 2.8)	0.5 (0.4, 0.7)
South Asia	18.5 (12.2, 27.2)	14.7 (5.9, 24.5)	7.8 (2.8, 13.1)	18.7 (6.8, 31.7)	5.6 (1.6, 9.4)	1.9 (0.8, 3.1)	1.9 (0.7, 3.5)	4.1 (1.7, 6.6)	2.1 (0.9, 3.8)	1.3 (0.2, 2.1)	1.5 (0.0, 4.4)	3.4 (2.2, 5.1)
Southeast Asia and Oceania	14.4 (9.5, 21.3)	17.9 (11.8, 26.3)	13.8 (5.0, 21.1)	7.6 (0.0, 12.8)	5.1 (2.7, 8.1)	4.3 (2.5, 6.6)	0.9 (0.5, 1.6)	2.1 (1.2, 3.3)	2.1 (0.2, 3.8)	0.0 (0.0, 0.0)	2.5 (0.0, 5.4)	0.0 (0.0, 0.0)
**2019-2020**												
Overall	15.9 (13.8, 18.3)	12.9 (10.1, 16.0)	6.9 (5.0, 8.8)	8.8 (6.3, 11.8)	4.0 (2.8, 5.2)	4.7 (3.6, 5.8)	2.6 (2.0, 3.3)	2.5 (1.8, 3.2)	2.1 (1.2, 3.0)	0.5 (0.3, 0.8)	1.5 (0.6, 2.6)	1.5 (1.1, 2.1)
Central America	21.0 (15.0, 29.4)	12.0 (7.8, 17.1)	22.6 (12.7, 32.0)	8.7 (3.5, 13.8)	4.1 (2.0, 6.0)	1.7 (1.0, 2.5)	0.4 (0.1, 0.8)	3.7 (2.0, 5.7)	1.7 (0.7, 2.6)	0.2 (0.0, 0.3)	0.2 (0.0, 0.6)	0.0 (0.0, 0.0)
South America	13.1 (9.6, 17.3)	13.8 (9.9, 19.0)	19.3 (14.4, 25.4)	6.5 (4.0, 9.1)	5.2 (3.4, 7.3)	3.4 (2.3, 4.7)	0.4 (0.3, 0.5)	1.3 (0.8, 1.7)	1.9 (1.2, 2.8)	0.3 (0.1, 0.5)	0.2 (0.1, 0.4)	0.0 (0.0, 0.0)
Eastern Europe	12.1 (7.8, 18.0)	5.7 (3.8, 8.2)	10.7 (6.9, 15.8)	0.9 (0.4, 1.4)	2.0 (1.3, 3.0)	1.0 (0.6, 1.7)	0.3 (0.1, 0.5)	0.3 (0.1, 0.5)	5.3 (2.2, 9.0)	1.4 (0.4, 2.6)	1.0 (0.0, 2.1)	0.9 (0.6, 1.4)
Central and Western Asia	22.0 (13.7, 32.3)	13.4 (8.7, 19.5)	11.4 (7.3, 16.5)	6.5 (2.7, 10.1)	5.5 (3.1, 8.2)	0.8 (0.5, 1.2)	1.8 (0.7, 2.9)	2.6 (1.6, 4.1)	0.9 (0.4, 1.4)	0.2 (0.0, 0.4)	1.5 (0.0, 3.0)	0.0 (0.0, 0.0)
West Africa	19.4 (15.7, 23.5)	8.0 (5.9, 10.1)	6.5 (5.0, 8.2)	3.7 (2.3, 5.2)	2.7 (2.0, 3.5)	2.8 (2.1, 3.5)	2.4 (1.6, 3.3)	2.1 (1.5, 2.8)	2.0 (1.3, 2.8)	0.6 (0.3, 0.9)	2.0 (0.7, 3.3)	0.1 (0.1, 0.2)
East and Southern Africa	17.3 (14.1, 21.0)	15.4 (11.1, 20.3)	7.4 (4.2, 10.0)	6.8 (3.3, 9.6)	4.3 (2.9, 6.2)	5.0 (3.7, 6.8)	3.5 (2.3, 4.7)	3.1 (1.8, 4.6)	2.2 (0.7, 3.4)	0.1 (0.0, 0.1)	1.2 (0.3, 2.4)	0.0 (0.0, 0.0)
South Asia	13.1 (9.5, 17.7)	12.5 (7.2, 18.4)	6.3 (3.0, 10.0)	12.8 (7.5, 19.7)	4.1 (1.7, 6.4)	5.2 (3.0, 7.4)	1.9 (1.1, 2.9)	2.1 (1.2, 3.0)	2.0 (0.5, 3.8)	1.0 (0.4, 1.6)	1.6 (0.0, 4.0)	3.5 (2.5, 4.9)
Southeast Asia and Oceania	11.1 (6.6, 17.0)	19.3 (12.0, 28.3)	5.5 (1.0, 8.6)	8.0 (0.0, 13.0)	0.0 (0.0, 0.0)	10.1 (6.2, 15.4)	3.1 (1.2, 5.2)	0.0 (0.0, 0.0)	0.0 (0.0, 0.0)	0.0 (0.0, 0.0)	2.4 (0.0, 5.8)	0.0 (0.0, 0.0)
**2021-2022**												
Overall	18.4 (15.8, 21.5)	16.4 (11.8, 21.1)	6.0 (4.4, 7.9)	5.7 (4.2, 7.3)	2.9 (1.9, 3.8)	3.7 (2.9, 4.6)	2.4 (1.8, 3.1)	1.2 (0.8, 1.5)	2.4 (1.5, 3.3)	1.5 (0.8, 2.2)	1.0 (0.5, 1.6)	1.8 (1.2, 2.5)
Central America	11.8 (8.8, 15.6)	9.5 (6.3, 13.2)	15.9 (7.2, 23.4)	6.1 (2.8, 9.3)	4.4 (2.2, 6.3)	2.7 (1.6, 3.9)	0.1 (0.0, 0.2)	2.2 (1.4, 3.3)	2.8 (1.1, 4.4)	1.2 (0.1, 2.1)	0.5 (0.0, 1.3)	0.0 (0.0, 0.0)
South America	8.7 (6.8, 11.4)	12.1 (9.0, 16.3)	21.4 (15.5, 28.8)	6.1 (4.1, 8.5)	3.2 (2.3, 4.4)	3.1 (2.2, 4.2)	0.2 (0.1, 0.3)	2.1 (1.4, 3.0)	3.6 (2.2, 5.1)	0.3 (0.1, 0.4)	0.1 (0.0, 0.2)	0.0 (0.0, 0.0)
Eastern Europe	23.9 (15.7, 35.2)	1.1 (0.8, 1.7)	12.3 (8.0, 18.2)	6.4 (3.4, 9.9)	0.6 (0.4, 1.0)	0.7 (0.5, 1.1)	0.1 (0.1, 0.2)	1.1 (0.6, 1.8)	2.9 (1.2, 5.1)	0.3 (0.0, 0.5)	0.3 (0.0, 0.6)	0.0 (0.0, 0.0)
Central and Western Asia	16.7 (11.1, 24.0)	9.6 (6.2, 14.0)	12.2 (7.6, 17.9)	4.1 (1.6, 6.2)	4.0 (2.6, 5.9)	1.3 (0.8, 1.9)	2.3 (1.1, 4.0)	0.4 (0.3, 0.6)	0.7 (0.3, 1.1)	0.0 (0.0, 0.1)	0.5 (0.0, 1.3)	0.0 (0.0, 0.0)
West Africa	26.0 (20.5, 32.5)	7.6 (5.0, 10.1)	8.3 (6.3, 10.7)	12.0 (6.2, 17.7)	2.3 (1.6, 3.2)	3.0 (2.2, 4.0)	2.5 (1.5, 3.6)	0.7 (0.5, 1.0)	2.2 (1.0, 3.3)	1.2 (0.6, 1.8)	1.4 (0.4, 2.6)	0.8 (0.5, 1.1)
East and Southern Africa	19.9 (16.5, 24.1)	18.3 (13.0, 24.8)	5.6 (3.7, 7.5)	5.1 (3.0, 7.1)	3.4 (2.2, 4.7)	5.3 (3.9, 6.9)	3.9 (2.6, 5.4)	1.4 (0.9, 2.0)	2.6 (1.3, 3.8)	1.2 (0.2, 2.1)	1.3 (0.4, 2.3)	0.0 (0.0, 0.0)
South Asia	15.0 (10.8, 20.9)	16.9 (7.8, 25.5)	5.6 (2.5, 9.4)	4.7 (2.5, 7.3)	2.6 (0.7, 4.2)	2.5 (1.3, 3.7)	1.0 (0.5, 1.7)	1.1 (0.5, 1.7)	2.3 (0.6, 4.0)	2.0 (0.5, 3.2)	0.6 (0.0, 1.5)	3.7 (2.6, 5.3)
Southeast Asia and Oceania	14.1 (9.1, 20.8)	12.3 (8.2, 17.9)	14.4 (5.2, 22.0)	15.3 (1.4, 27.0)	3.5 (1.6, 5.7)	0.0 (0.0, 0.0)	4.5 (2.1, 7.9)	0.0 (0.0, 0.0)	3.4 (1.0, 6.0)	0.0 (0.0, 0.0)	0.1 (0.0, 0.2)	0.0 (0.0, 0.0)

Attributable fractions are expressed as a percent. tEPEC=typical enteropathogenic *E. coli*. ETEC=heat-stable enterotoxigenic *E. coli*

Table C. Pathogen-specific attributable fractions with 95% confidence intervals of diarrhea hospitalizations in children aged <5 years by Global Pediatric Diarrhea Surveillance site, 2021-2022 (Underlying data for Figure 5).

Geographic Grouping	Surveillance Site	Rotavirus	*Shigella*	Norovirus	Adenovirus 40/41	Sapovirus	ETEC	*Cryptosporidium*	Astrovirus	*C. jejuni/coli*	*Salmonella*	tEPEC	*V. cholerae*
Central America	Honduras	10.3 (8.8, 10.9)	7.7 (4.7, 9.5)	20.1 (7.8, 23.6)	7.7 (3.2, 10.1)	4.0 (0.9, 5.2)	2.9 (1.5, 3.7)	0.1 (0.1, 0.2)	2.2 (1.3, 2.9)	2.6 (0.0, 3.7)	0.0 (0.0, 0.0)	0.4 (0.0, 1.3)	0.0 (0.0, 0.0)
	Nicaragua	15.4 (13.5, 16.9)	13.8 (10.0, 16.0)	10.3 (8.1, 11.8)	3.5 (0.7, 4.7)	5.6 (4.4, 6.6)	2.8 (1.8, 3.3)	0.0 (0.0, 0.0)	2.6 (1.6, 3.2)	3.9 (1.5, 5.6)	3.9 (0.7, 5.5)	0.7 (0.0, 1.7)	0.0 (0.0, 0.0)
South America	Bolivia	11.7 (9.5, 12.8)	15.7 (11.9, 18.0)	23.7 (9.3, 26.5)	5.4 (2.2, 7.6)	3.7 (0.6, 4.5)	6.0 (2.8, 7.3)	0.6 (0.3, 0.9)	1.4 (0.6, 1.9)	6.8 (1.0, 10.4)	0.3 (0.1, 0.6)	0.1 (0.0, 0.2)	0.0 (0.0, 0.0)
	Ecuador	6.9 (5.5, 8.0)	6.2 (5.1, 6.9)	22.1 (17.5, 24.8)	9.4 (4.5, 12.3)	3.2 (2.4, 3.7)	0.9 (0.7, 1.0)	0.1 (0.1, 0.2)	1.5 (0.9, 1.9)	3.9 (1.0, 5.5)	0.9 (0.1, 1.3)	0.0 (0.0, 0.0)	0.0 (0.0, 0.0)
	Peru	7.7 (6.4, 8.7)	12.9 (9.7, 14.3)	23.1 (17.3, 26.3)	6.4 (4.1, 7.8)	3.2 (2.2, 3.9)	3.0 (2.3, 3.4)	0.1 (0.1, 0.2)	2.6 (1.7, 3.2)	2.6 (1.1, 3.8)	0.0 (0.0, 0.0)	0.1 (0.0, 0.2)	0.0 (0.0, 0.0)
	Paraguay	14.4 (10.8, 16.2)	10.4 (8.9, 11.6)	11.6 (8.3, 13.1)	3.0 (1.1, 3.4)	2.8 (2.0, 3.4)	2.0 (1.5, 2.4)	0.2 (0.1, 0.2)	1.2 (0.8, 1.5)	4.7 (1.7, 6.9)	1.4 (0.0, 1.8)	0.6 (0.0, 1.1)	0.0 (0.0, 0.0)
Eastern Europe	Moldova	24.6 (19.7, 26.4)	1.2 (1.1, 1.2)	12.6 (10.1, 13.9)	6.6 (4.3, 8.1)	0.6 (0.4, 0.8)	0.7 (0.6, 0.9)	0.1 (0.1, 0.2)	1.2 (0.7, 1.5)	3.1 (1.2, 4.5)	0.3 (0.0, 0.5)	0.3 (0.0, 0.6)	0.0 (0.0, 0.0)
	Ukraine*	---	---	---	---	---	---	---	---	---	---	---	---
Central and Western Asia	Armenia	13.8 (11.3, 15.9)	2.3 (1.9, 2.6)	8.7 (7.3, 10.1)	4.9 (2.3, 6.0)	2.6 (2.0, 2.9)	1.3 (1.0, 1.5)	0.0 (0.0, 0.0)	4.9 (3.6, 6.2)	4.3 (2.3, 6.4)	1.0 (0.1, 1.4)	0.3 (0.0, 0.6)	0.0 (0.0, 0.0)
	Tajikistan	17.1 (14.0, 19.8)	10.2 (8.2, 11.7)	12.8 (9.3, 15.0)	4.3 (1.5, 5.2)	4.2 (3.3, 5.0)	1.3 (1.0, 1.6)	2.4 (1.2, 3.6)	0.2 (0.1, 0.3)	0.6 (0.2, 0.8)	0.0 (0.0, 0.0)	0.6 (0.0, 1.2)	0.0 (0.0, 0.0)
	Uzbekistan*	---	---	---	---	---	---	---	---	---	---	---	---
West Africa	Benin	23.6 (19.8, 24.8)	12.5 (4.7, 13.6)	3.5 (1.4, 6.0)	14.1 (4.7, 18.4)	0.0 (0.0, 0.0)	3.0 (1.3, 4.7)	1.8 (0.7, 2.4)	0.1 (0.0, 0.1)	2.6 (0.3, 3.5)	0.0 (0.0, 0.0)	0.0 (0.0, 1.4)	3.5 (3.4, 3.7)
	Burkina Faso	20.4 (15.6, 23.1)	4.8 (3.6, 5.6)	7.9 (6.2, 8.8)	9.1 (0.0, 12.2)	3.1 (1.9, 3.8)	3.1 (2.2, 3.8)	3.4 (2.0, 4.8)	1.0 (0.6, 1.3)	3.0 (0.3, 4.3)	0.8 (0.1, 1.2)	1.7 (0.0, 3.7)	0.0 (0.0, 0.0)
	Côte d'Ivoire*	---	---	---	---	---	---	---	---	---	---	---	---
	Ghana	37.6 (31.3, 41.7)	10.1 (3.6, 12.5)	12.3 (9.5, 13.5)	17.0 (1.2, 26.9)	3.0 (2.0, 3.6)	3.1 (2.3, 3.7)	1.8 (0.7, 2.5)	0.7 (0.4, 0.8)	1.1 (0.3, 1.8)	2.7 (1.2, 3.8)	1.7 (0.0, 3.0)	0.0 (0.0, 0.0)
	Nigeria	38.2 (30.5, 42.0)	4.4 (2.0, 5.5)	7.4 (3.0, 8.6)	9.1 (3.5, 13.1)	2.2 (0.5, 2.9)	1.3 (0.5, 1.8)	1.8 (0.6, 3.3)	1.3 (0.6, 1.6)	0.1 (0.0, 0.1)	0.2 (0.0, 0.3)	0.4 (0.0, 1.0)	0.0 (0.0, 0.0)
East and Southern Africa	Ethiopia	11.2 (8.8, 12.3)	22.6 (16.0, 26.1)	5.8 (2.7, 6.8)	4.0 (0.6, 6.1)	4.3 (2.5, 5.1)	4.4 (3.3, 5.5)	2.7 (1.1, 4.0)	1.7 (1.0, 2.1)	2.1 (0.1, 3.3)	0.2 (0.0, 0.4)	1.0 (0.0, 2.1)	0.0 (0.0, 0.0)
	Madagascar	35.1 (27.9, 37.9)	24.5 (16.9, 28.7)	4.0 (1.2, 5.4)	5.5 (0.3, 8.2)	3.4 (0.7, 4.6)	12.4 (7.2, 15.0)	6.7 (3.3, 9.3)	1.7 (0.7, 2.1)	5.6 (2.0, 8.2)	5.7 (0.0, 8.8)	1.3 (0.0, 3.5)	0.0 (0.0, 0.0)
	Mauritius	10.2 (7.7, 12.2)	2.9 (2.2, 3.4)	14.9 (9.9, 17.0)	16.6 (2.4, 25.8)	3.8 (2.9, 4.5)	0.4 (0.3, 0.5)	0.3 (0.2, 0.5)	0.9 (0.5, 1.2)	7.4 (1.4, 10.1)	2.8 (0.0, 4.4)	0.8 (0.0, 1.4)	0.0 (0.0, 0.0)
	Uganda	28.3 (22.3, 31.1)	5.4 (4.3, 6.2)	6.4 (4.0, 7.7)	3.1 (0.0, 5.3)	1.7 (1.3, 2.1)	2.7 (1.8, 3.5)	7.7 (3.9, 10.0)	0.1 (0.1, 0.1)	1.6 (0.1, 3.0)	1.1 (0.0, 2.5)	2.4 (0.1, 4.7)	0.0 (0.0, 0.0)
	Zambia	47.7 (38.9, 50.6)	6.5 (4.8, 8.1)	7.9 (3.3, 15.1)	15.9 (12.8, 24.5)	1.3 (0.0, 2.9)	3.3 (1.9, 4.5)	2.1 (1.3, 2.4)	1.7 (0.0, 2.6)	1.5 (0.0, 2.8)	1.4 (0.0, 1.7)	0.0 (0.0, 3.4)	0.0 (0.0, 0.0)
	Zimbabwe	22.4 (18.3, 24.5)	13.8 (9.4, 15.4)	7.1 (5.5, 7.9)	11.4 (0.4, 18.0)	3.1 (2.1, 4.1)	6.7 (4.6, 8.2)	0.9 (0.5, 1.3)	1.9 (1.2, 2.4)	4.6 (2.1, 6.2)	0.6 (0.3, 0.8)	3.3 (0.0, 5.9)	0.0 (0.0, 0.0)
South Asia	India #1	17.8 (14.6, 19.4)	5.2 (1.8, 6.3)	7.4 (2.2, 8.7)	16.7 (7.0, 25.0)	2.0 (0.0, 2.7)	0.3 (0.1, 0.4)	1.0 (0.5, 1.5)	3.0 (0.9, 3.9)	2.4 (0.4, 3.4)	0.0 (0.0, 0.0)	0.1 (0.0, 0.5)	2.7 (2.6, 2.8)
	India #2*	---	---	---	---	---	---	---	---	---	---	---	---
	India #3	14.6 (10.9, 16.0)	13.9 (5.0, 18.2)	6.6 (2.3, 7.9)	11.2 (5.3, 15.6)	2.1 (0.3, 2.9)	2.2 (0.7, 2.7)	3.5 (1.7, 5.5)	2.0 (0.6, 2.7)	2.3 (0.3, 3.5)	0.3 (0.1, 0.4)	0.8 (0.0, 6.1)	2.8 (2.2, 3.1)
	India #4	5.6 (4.2, 6.4)	7.0 (3.4, 8.9)	11.4 (4.1, 13.9)	11.3 (1.5, 16.4)	8.9 (1.1, 11.8)	1.7 (0.7, 2.2)	0.0 (0.0, 0.0)	4.1 (1.5, 5.5)	1.5 (0.4, 2.3)	0.0 (0.0, 0.0)	0.2 (0.0, 0.6)	0.0 (0.0, 0.0)
	India #5	17.8 (15.6, 18.8)	20.0 (7.1, 24.4)	7.6 (3.0, 9.5)	5.7 (3.1, 7.7)	3.1 (0.2, 4.0)	0.5 (0.2, 0.7)	0.0 (0.0, 0.0)	0.7 (0.2, 0.9)	1.9 (0.4, 3.1)	2.6 (0.8, 3.3)	0.1 (0.0, 0.7)	0.0 (0.0, 0.0)
	Pakistan #1	6.9 (5.3, 8.0)	14.8 (10.5, 17.8)	2.9 (0.9, 4.1)	3.1 (0.0, 4.9)	3.2 (0.0, 5.3)	10.5 (4.5, 12.9)	5.2 (2.6, 7.5)	3.0 (0.3, 4.4)	4.0 (0.0, 7.2)	1.4 (0.0, 2.0)	2.4 (0.0, 6.0)	9.8 (8.3, 10.5)
	Pakistan #2	10.6 (7.9, 11.9)	6.8 (4.9, 8.0)	2.3 (0.3, 4.3)	2.3 (0.2, 3.7)	0.9 (0.0, 1.3)	5.6 (2.5, 7.0)	2.0 (0.9, 2.9)	1.7 (0.1, 2.6)	3.2 (0.0, 6.2)	0.6 (0.0, 0.9)	1.1 (0.0, 2.9)	17.9 (16.1, 18.7)
Southeast Asia and Oceania	Fiji	14.5 (10.9, 16.2)	12.6 (10.9, 13.3)	15.5 (5.6, 16.7)	15.5 (1.7, 22.4)	3.6 (1.7, 4.6)	0.0 (0.0, 0.0)	4.5 (2.8, 6.8)	0.0 (0.0, 0.0)	3.7 (1.1, 5.1)	0.0 (0.0, 0.0)	0.1 (0.0, 0.2)	0.0 (0.0, 0.0)
	Indonesia*	---	---	---	---	---	---	---	---	---	---	---	---
	Lao PDR	19.3 (16.1, 21.3)	3.4 (2.5, 3.8)	17.7 (15.1, 19.2)	6.3 (1.2, 8.9)	3.8 (2.5, 4.7)	2.2 (1.6, 2.6)	0.4 (0.2, 0.6)	0.7 (0.4, 0.8)	2.3 (1.0, 3.1)	2.2 (0.9, 3.1)	0.0 (0.0, 0.1)	0.0 (0.0, 0.0)
	Myanmar*	---	---	---	---	---	---	---	---	---	---	---	---
	Philippines	22.9 (17.0, 24.5)	8.5 (5.8, 10.9)	17.1 (8.1, 19.6)	3.3 (0.0, 4.9)	4.4 (2.3, 5.2)	2.1 (1.1, 2.5)	0.2 (0.1, 0.3)	0.9 (0.5, 1.1)	7.0 (3.2, 9.5)	3.5 (1.7, 4.3)	0.8 (0.0, 1.5)	12.6 (11.3, 13.0)
	Viet Nam	18.4 (13.7, 21.9)	0.7 (0.6, 0.8)	16.3 (13.2, 18.8)	1.8 (0.5, 2.5)	1.2 (0.8, 1.4)	0.0 (0.0, 0.0)	0.0 (0.0, 0.0)	3.6 (2.1, 4.6)	0.7 (0.2, 0.9)	0.5 (0.0, 0.8)	0.0 (0.0, 0.0)	0.0 (0.0, 0.0)
East Asia	China #1	7.1 (5.3, 8.1)	0.0 (0.0, 0.0)	1.7 (1.4, 2.0)	2.8 (1.5, 3.3)	0.1 (0.0, 0.1)	0.0 (0.0, 0.0)	0.0 (0.0, 0.0)	0.5 (0.3, 0.7)	0.1 (0.0, 0.1)	0.4 (0.0, 0.7)	0.0 (0.0, 0.0)	0.0 (0.0, 0.0)
	China #2	20.8 (15.1, 23.0)	0.0 (0.0, 0.0)	9.5 (7.7, 10.5)	9.4 (2.3, 12.7)	1.0 (0.8, 1.2)	0.0 (0.0, 0.0)	0.0 (0.0, 0.0)	1.8 (1.1, 2.1)	0.9 (0.2, 1.2)	6.3 (1.0, 9.0)	0.0 (0.0, 0.0)	0.0 (0.0, 0.0)
	China #3	18.7 (15.2, 20.7)	0.0 (0.0, 0.0)	18.0 (14.5, 19.8)	5.1 (2.9, 6.9)	0.4 (0.3, 0.5)	0.0 (0.0, 0.0)	0.2 (0.1, 0.3)	0.4 (0.2, 0.5)	0.2 (0.1, 0.4)	1.2 (0.3, 1.8)	0.4 (0.0, 0.8)	0.0 (0.0, 0.0)

Attributable fractions are expressed as a percent. Lao PDR=Lao People's Democratic Republic. tEPEC=typical enteropathogenic *E. coli*. ETEC=enterotoxigenic *E. coli*.
*Site did not participate in GPDS during this two-year time period

Table D. Pathogen-specific attributable fractions with 95% confidence intervals of diarrhea hospitalizations in children aged <5 years by Global Pediatric Diarrhea Surveillance site, 2019-2020 (Underlying data for Figure 5).

Geographic Grouping	Surveillance Site	Rotavirus	*Shigella*	Norovirus	Adenovirus 40/41	Sapovirus	ETEC	*Cryptosporidium*	Astrovirus	*C. jejuni/coli*	*Salmonella*	tEPEC	*V. cholerae*
Central America	Honduras	25.2 (22.2, 27.1)	11.4 (6.7, 14.4)	20.7 (5.6, 24.3)	9.8 (3.2, 14.5)	3.9 (0.9, 4.9)	1.8 (0.7, 2.3)	0.6 (0.3, 1.0)	4.1 (1.9, 5.6)	1.5 (0.0, 2.4)	0.1 (0.0, 0.1)	0.2 (0.0, 0.5)	0.0 (0.0, 0.0)
	Nicaragua	14.1 (11.2, 15.5)	13.8 (10.7, 15.3)	31.3 (25.7, 35.0)	7.3 (1.8, 11.8)	5.0 (3.4, 6.3)	1.9 (1.4, 2.2)	0.0 (0.0, 0.0)	3.2 (1.6, 4.2)	2.2 (0.7, 3.0)	0.4 (0.0, 0.6)	0.4 (0.0, 0.7)	0.0 (0.0, 0.0)
South America	Bolivia	17.1 (14.0, 18.6)	15.6 (11.5, 18.1)	26.2 (10.0, 30.4)	9.9 (2.5, 13.9)	3.3 (0.4, 4.2)	6.1 (2.9, 7.6)	0.4 (0.1, 0.6)	2.5 (1.1, 3.3)	2.4 (1.2, 3.3)	0.7 (0.2, 0.9)	0.2 (0.0, 0.7)	0.0 (0.0, 0.0)
	Ecuador	12.5 (9.8, 13.9)	7.8 (6.5, 8.5)	29.0 (23.2, 31.8)	7.2 (2.4, 9.3)	3.9 (3.0, 4.8)	1.5 (1.1, 1.7)	1.1 (0.6, 1.7)	1.9 (1.1, 2.4)	2.6 (1.0, 4.1)	0.5 (0.1, 0.7)	0.2 (0.0, 0.4)	0.0 (0.0, 0.0)
	Peru	13.5 (10.0, 14.9)	15.9 (12.7, 18.1)	17.9 (14.1, 20.2)	5.8 (3.8, 7.5)	6.1 (4.1, 7.4)	3.6 (2.4, 4.3)	0.2 (0.1, 0.3)	1.1 (0.6, 1.4)	1.5 (0.6, 2.4)	0.2 (0.0, 0.4)	0.2 (0.0, 0.4)	0.0 (0.0, 0.0)
	Paraguay	6.1 (4.9, 6.9)	5.1 (3.9, 6.1)	12.1 (8.8, 13.5)	9.3 (3.1, 12.9)	3.8 (2.7, 4.6)	0.4 (0.3, 0.5)	1.0 (0.4, 1.4)	0.2 (0.1, 0.3)	4.3 (1.4, 6.0)	0.1 (0.0, 0.1)	0.6 (0.0, 1.5)	0.0 (0.0, 0.0)
Eastern Europe	Moldova	12.6 (9.3, 13.8)	5.8 (5.1, 6.4)	11.0 (8.9, 12.4)	0.9 (0.6, 1.2)	2.1 (1.7, 2.4)	1.0 (0.6, 1.4)	0.3 (0.1, 0.4)	0.3 (0.1, 0.4)	5.5 (2.8, 7.7)	1.5 (0.5, 2.2)	1.0 (0.0, 1.9)	1.0 (0.8, 1.0)
	Ukraine	26.2 (21.4, 28.9)	0.0 (0.0, 0.0)	12.7 (11.0, 14.0)	9.7 (4.6, 12.5)	1.7 (1.1, 2.0)	0.0 (0.0, 0.0)	0.0 (0.0, 0.0)	0.0 (0.0, 0.0)	7.0 (2.9, 9.4)	1.7 (0.4, 2.6)	0.2 (0.0, 0.4)	0.0 (0.0, 0.0)
Central and Western Asia	Armenia	5.4 (4.2, 6.2)	4.5 (4.0, 4.8)	4.1 (3.4, 4.7)	11.7 (5.1, 16.3)	1.2 (0.8, 1.5)	2.9 (2.0, 3.7)	0.0 (0.0, 0.0)	0.0 (0.0, 0.0)	4.9 (2.7, 7.0)	4.6 (0.7, 6.6)	0.0 (0.0, 0.0)	0.0 (0.0, 0.0)
	Tajikistan	23.6 (17.6, 26.4)	14.2 (11.0, 16.5)	12.1 (8.9, 13.6)	6.6 (2.8, 8.5)	5.9 (3.5, 7.0)	0.7 (0.5, 0.9)	1.9 (0.8, 2.5)	2.8 (2.1, 3.6)	0.7 (0.2, 1.0)	0.0 (0.0, 0.0)	1.6 (0.0, 2.9)	0.0 (0.0, 0.0)
	Uzbekistan*	---	---	---	---	---	---	---	---	---	---	---	---
West Africa	Benin	36.9 (30.6, 38.6)	19.2 (10.0, 20.1)	3.8 (1.7, 6.8)	7.3 (1.0, 10.9)	2.1 (0.3, 3.4)	3.8 (1.5, 5.5)	3.5 (1.4, 4.4)	1.6 (0.4, 2.1)	4.7 (1.8, 5.9)	0.4 (0.2, 0.5)	0.0 (0.0, 3.2)	0.9 (0.9, 0.9)
	Burkina Faso	15.7 (11.9, 17.6)	4.6 (3.7, 5.4)	4.3 (3.5, 4.8)	2.3 (1.2, 3.0)	5.1 (3.9, 6.3)	1.5 (1.2, 1.9)	0.7 (0.4, 1.0)	3.7 (2.4, 4.6)	2.5 (1.0, 3.5)	0.3 (0.0, 0.5)	2.3 (0.0, 4.1)	0.0 (0.0, 0.0)
	Côte d'Ivoire	10.1 (7.4, 11.4)	5.9 (4.4, 6.8)	6.7 (4.4, 8.7)	1.2 (0.0, 2.2)	0.8 (0.6, 1.0)	2.2 (1.6, 2.9)	3.3 (1.5, 4.7)	2.0 (1.0, 2.6)	1.0 (0.2, 1.8)	0.6 (0.0, 1.4)	3.2 (0.0, 5.8)	0.0 (0.0, 0.0)
	Ghana	26.0 (22.7, 28.2)	9.1 (3.6, 11.9)	10.9 (8.2, 12.2)	6.9 (2.4, 10.1)	2.4 (1.8, 2.8)	4.5 (3.1, 5.2)	2.9 (1.5, 4.3)	0.6 (0.5, 0.9)	1.5 (0.3, 2.3)	1.0 (0.3, 1.3)	1.2 (0.0, 2.2)	0.0 (0.0, 0.0)
	Nigeria	51.9 (37.3, 57.0)	5.4 (2.6, 6.8)	6.5 (2.5, 7.5)	16.1 (3.7, 23.0)	4.1 (0.6, 5.5)	2.3 (1.0, 2.7)	1.4 (0.6, 2.3)	1.0 (0.4, 1.3)	0.6 (0.2, 0.8)	0.7 (0.2, 0.9)	0.7 (0.0, 1.9)	0.0 (0.0, 0.0)
East and Southern Africa	Ethiopia	11.0 (9.0, 13.0)	17.8 (11.7, 20.2)	9.0 (3.9, 10.6)	5.9 (0.0, 8.7)	6.2 (4.1, 7.3)	5.7 (4.1, 6.8)	2.1 (0.9, 3.3)	4.8 (2.6, 5.9)	2.6 (0.0, 3.9)	0.0 (0.0, 0.0)	1.3 (0.0, 3.0)	0.0 (0.0, 0.0)
	Madagascar	23.3 (17.7, 25.7)	17.9 (10.9, 21.1)	6.4 (1.9, 8.4)	9.9 (1.7, 14.7)	1.8 (0.6, 2.6)	5.8 (2.9, 7.5)	4.9 (1.9, 6.5)	1.0 (0.6, 1.3)	1.3 (0.4, 1.8)	0.1 (0.0, 0.3)	1.2 (0.0, 2.6)	0.0 (0.0, 0.0)
	Mauritius	6.7 (5.0, 7.6)	1.4 (1.2, 1.5)	7.0 (4.4, 7.8)	9.9 (1.2, 13.7)	4.5 (3.3, 5.3)	0.2 (0.1, 0.3)	0.0 (0.0, 0.0)	0.0 (0.0, 0.0)	4.1 (1.1, 6.3)	1.9 (0.0, 2.8)	1.4 (0.0, 2.6)	0.0 (0.0, 0.0)
	Uganda	17.8 (13.6, 19.9)	5.1 (3.8, 6.0)	6.3 (3.9, 7.7)	3.9 (0.0, 6.5)	1.8 (1.5, 2.2)	4.2 (2.9, 5.2)	7.3 (3.3, 10.1)	1.0 (0.6, 1.4)	1.9 (0.2, 3.8)	0.0 (0.0, 0.0)	1.3 (0.0, 2.6)	0.0 (0.0, 0.0)
	Zambia	48.7 (38.7, 52.0)	24.2 (18.6, 28.4)	6.0 (2.6, 12.1)	19.9 (14.6, 30.9)	2.5 (0.0, 7.4)	4.5 (2.3, 6.2)	4.6 (2.0, 5.5)	1.4 (0.0, 2.4)	1.8 (0.0, 3.1)	0.0 (0.0, 0.0)	0.0 (0.0, 5.6)	0.0 (0.0, 0.0)
	Zimbabwe	34.6 (27.3, 38.2)	11.6 (7.0, 13.7)	0.8 (0.7, 0.9)	3.6 (0.2, 5.1)	2.5 (1.8, 3.1)	1.8 (1.4, 2.2)	2.0 (1.0, 2.8)	1.5 (1.1, 2.0)	3.7 (1.5, 5.4)	1.7 (0.4, 2.3)	2.2 (0.0, 3.9)	0.0 (0.0, 0.0)
South Asia	India #1	17.7 (14.3, 19.0)	20.5 (9.0, 24.9)	7.2 (2.4, 8.8)	25.6 (13.8, 36.0)	3.2 (0.2, 4.3)	3.4 (1.6, 4.1)	0.5 (0.2, 0.7)	3.0 (1.3, 3.8)	1.6 (0.1, 2.4)	1.2 (0.1, 1.6)	1.0 (0.0, 2.4)	1.4 (0.9, 1.6)
	India #2*	---	---	---	---	---	---	---	---	---	---	---	---
	India #3	12.3 (10.5, 13.6)	11.6 (4.9, 15.6)	8.1 (2.4, 9.5)	18.8 (2.8, 27.7)	6.7 (1.0, 9.3)	2.9 (1.3, 3.5)	2.0 (1.0, 3.2)	2.9 (1.6, 3.9)	1.8 (0.3, 2.5)	0.6 (0.2, 0.8)	0.7 (0.0, 4.5)	0.4 (0.3, 0.4)
	India #4	10.8 (8.2, 12.3)	7.2 (2.6, 9.6)	10.0 (3.5, 12.3)	6.7 (2.1, 10.4)	3.9 (0.7, 5.5)	3.1 (1.2, 4.3)	1.3 (0.5, 2.2)	1.8 (0.5, 2.3)	0.9 (0.1, 1.4)	0.9 (0.1, 1.3)	0.6 (0.0, 3.0)	0.0 (0.0, 0.0)
	India #5*	---	---	---	---	---	---	---	---	---	---	---	---
	Pakistan #1	12.9 (10.1, 14.2)	12.7 (9.1, 15.0)	4.0 (0.8, 6.3)	5.3 (0.0, 8.4)	4.0 (0.2, 5.8)	10.6 (4.0, 13.0)	3.3 (1.4, 4.8)	1.2 (0.3, 1.9)	3.9 (0.0, 7.7)	1.4 (0.0, 2.2)	2.7 (0.0, 8.1)	10.4 (9.5, 10.8)
	Pakistan #2*	---	---	---	---	---	---	---	---	---	---	---	---
Southeast Asia and Oceania	Fiji	11.6 (7.5, 13.4)	19.7 (14.6, 21.8)	5.9 (1.2, 6.6)	8.6 (0.0, 10.2)	0.0 (0.0, 0.0)	10.5 (7.2, 12.1)	3.1 (1.5, 4.5)	0.0 (0.0, 0.0)	0.0 (0.0, 0.0)	0.0 (0.0, 0.0)	2.4 (0.0, 5.2)	0.0 (0.0, 0.0)
	Indonesia*	---	---	---	---	---	---	---	---	---	---	---	---
	Lao PDR	46.0 (34.9, 51.5)	4.5 (3.3, 4.8)	11.4 (9.4, 12.8)	2.8 (1.0, 3.7)	1.9 (1.2, 2.4)	1.5 (1.2, 1.8)	0.7 (0.4, 1.1)	0.0 (0.0, 0.0)	3.0 (1.0, 4.2)	4.4 (0.9, 5.7)	0.2 (0.0, 0.3)	0.0 (0.0, 0.0)
	Myanmar	60.4 (53.9, 63.6)	5.7 (4.5, 8.4)	7.4 (3.9, 10.8)	23.6 (1.3, 29.5)	1.8 (0.0, 2.8)	1.7 (0.6, 2.7)	0.4 (0.3, 0.6)	0.7 (0.0, 1.1)	1.8 (0.0, 4.9)	1.4 (0.0, 1.7)	0.0 (0.0, 2.7)	0.0 (0.0, 0.0)
	Philippines*	---	---	---	---	---	---	---	---	---	---	---	---
	Viet Nam	8.2 (6.2, 10.0)	0.4 (0.3, 0.5)	8.9 (5.8, 10.5)	0.1 (0.0, 0.2)	1.3 (0.9, 1.6)	0.0 (0.0, 0.0)	0.0 (0.0, 0.0)	0.7 (0.4, 0.8)	1.1 (0.3, 1.5)	0.5 (0.0, 0.9)	0.0 (0.0, 0.1)	0.0 (0.0, 0.0)
East Asia	China #1	9.1 (7.3, 10.6)	0.0 (0.0, 0.0)	7.3 (6.0, 8.1)	0.6 (0.3, 0.9)	0.9 (0.6, 1.1)	0.0 (0.0, 0.0)	0.0 (0.0, 0.0)	0.5 (0.3, 0.6)	0.6 (0.2, 0.9)	0.2 (0.0, 0.3)	0.0 (0.0, 0.0)	0.0 (0.0, 0.0)
	China #2	13.6 (9.9, 15.0)	0.0 (0.0, 0.0)	11.5 (9.8, 12.3)	2.6 (1.2, 3.4)	0.5 (0.3, 0.6)	0.0 (0.0, 0.0)	0.0 (0.0, 0.0)	1.1 (0.7, 1.3)	1.7 (0.6, 2.5)	2.0 (0.2, 3.1)	0.2 (0.0, 0.4)	0.0 (0.0, 0.0)
	China #3	38.9 (31.8, 43.3)	0.0 (0.0, 0.0)	22.4 (18.4, 24.6)	4.1 (1.6, 5.6)	2.5 (1.8, 3.0)	0.0 (0.0, 0.0)	0.4 (0.2, 0.5)	0.5 (0.3, 0.6)	0.0 (0.0, 0.0)	0.3 (0.0, 0.5)	0.3 (0.0, 0.5)	0.0 (0.0, 0.0)

Attributable fractions are expressed as a percent. Lao PDR=Lao People's Democratic Republic. tEPEC=typical enteropathogenic *E. coli*. ETEC=enterotoxigenic *E. coli*.
*Site did not participate in GPDS during this two-year time period

Table E. Pathogen-specific attributable fractions with 95% confidence intervals of diarrhea hospitalizations in children aged <5 years by Global Pediatric Diarrhea Surveillance site, 2017-2018 (Underlying data for Figure 5).

Geographic Grouping	Surveillance Site	Rotavirus	*Shigella*	Norovirus	Adenovirus 40/41	Sapovirus	ETEC	*Cryptosporidium*	Astrovirus	*C. jejuni/coli*	*Salmonella*	tEPEC	*V. cholerae*
Central America	Honduras	23.2 (20.3, 25.2)	20.8 (12.1, 26.4)	16.7 (5.9, 20.1)	11.7 (4.3, 16.1)	3.3 (0.5, 4.4)	3.7 (1.8, 4.5)	1.7 (0.7, 2.6)	5.5 (2.2, 7.5)	4.7 (0.3, 7.1)	0.4 (0.1, 0.7)	1.0 (0.0, 3.0)	0.0 (0.0, 0.0)
	Nicaragua	15.0 (11.4, 16.9)	10.9 (7.2, 13.2)	12.7 (9.8, 14.4)	5.0 (1.0, 7.1)	4.1 (2.9, 5.0)	0.8 (0.6, 1.0)	0.6 (0.3, 0.9)	4.8 (2.8, 6.0)	0.6 (0.2, 0.8)	0.7 (0.2, 0.9)	0.3 (0.0, 0.4)	0.0 (0.0, 0.0)
South America	Bolivia	19.9 (15.6, 22.6)	12.1 (9.2, 14.2)	24.1 (9.9, 27.4)	1.7 (0.4, 3.0)	3.9 (0.8, 4.7)	7.0 (2.3, 8.2)	1.6 (0.6, 2.6)	1.8 (0.8, 2.4)	3.8 (1.7, 5.3)	0.0 (0.0, 0.0)	0.2 (0.0, 0.5)	0.0 (0.0, 0.0)
	Ecuador	7.5 (5.8, 8.4)	5.4 (4.5, 5.8)	25.3 (19.5, 27.8)	6.1 (1.7, 8.5)	4.8 (3.5, 5.6)	2.7 (2.1, 3.2)	0.7 (0.4, 0.9)	3.1 (2.1, 3.8)	3.4 (1.6, 4.9)	1.2 (0.2, 1.8)	0.4 (0.0, 0.7)	0.0 (0.0, 0.0)
	Peru	13.6 (9.8, 15.4)	13.8 (11.6, 15.3)	25.1 (19.6, 27.9)	5.8 (3.5, 7.6)	4.6 (3.7, 5.1)	1.8 (1.3, 2.1)	0.3 (0.2, 0.4)	2.2 (1.3, 2.7)	2.7 (1.2, 3.7)	1.0 (0.3, 1.6)	0.2 (0.0, 0.3)	0.0 (0.0, 0.0)
	Paraguay	7.5 (4.9, 8.4)	13.6 (10.4, 15.0)	15.8 (12.1, 18.0)	11.8 (3.4, 15.7)	2.0 (1.7, 2.3)	1.3 (0.9, 1.7)	0.8 (0.4, 1.2)	1.0 (0.6, 1.3)	1.4 (0.7, 1.8)	0.0 (0.0, 0.0)	0.8 (0.0, 1.4)	0.0 (0.0, 0.0)
Eastern Europe	Moldova	19.4 (15.3, 21.0)	6.9 (5.9, 7.4)	9.6 (7.9, 10.8)	7.3 (3.3, 8.7)	3.0 (2.2, 3.5)	1.4 (1.1, 1.6)	1.0 (0.5, 1.4)	0.5 (0.3, 0.6)	4.4 (2.1, 6.5)	2.4 (0.6, 3.2)	0.1 (0.0, 0.2)	0.0 (0.0, 0.0)
	Ukraine	36.9 (30.3, 40.0)	0.3 (0.3, 0.4)	7.8 (6.7, 8.5)	1.3 (0.6, 1.9)	3.0 (2.1, 3.5)	0.8 (0.6, 0.9)	0.0 (0.0, 0.0)	0.8 (0.4, 0.9)	4.7 (2.1, 6.5)	1.7 (0.2, 2.5)	0.4 (0.0, 0.9)	0.0 (0.0, 0.0)
Central and Western Asia	Armenia	11.9 (9.7, 13.4)	8.7 (7.7, 9.5)	8.5 (6.3, 9.5)	2.0 (1.0, 2.3)	1.7 (1.1, 2.0)	3.1 (2.2, 3.7)	0.2 (0.2, 0.3)	1.6 (1.0, 2.0)	6.0 (2.4, 8.3)	0.5 (0.1, 0.7)	0.0 (0.0, 0.1)	0.0 (0.0, 0.0)
	Tajikistan	25.5 (19.7, 28.6)	12.5 (10.2, 14.3)	8.0 (5.5, 9.1)	3.6 (1.5, 5.1)	6.9 (5.4, 8.0)	3.9 (3.1, 4.7)	4.0 (2.2, 5.7)	4.3 (3.1, 5.3)	0.4 (0.1, 0.6)	0.0 (0.0, 0.0)	0.4 (0.0, 0.9)	0.0 (0.0, 0.0)
	Uzbekistan	10.2 (7.5, 11.1)	6.2 (5.0, 7.0)	15.7 (11.2, 19.0)	3.1 (0.0, 5.2)	5.3 (4.1, 6.2)	3.8 (2.7, 4.7)	0.9 (0.4, 1.3)	1.2 (0.8, 1.7)	0.2 (0.0, 0.4)	0.2 (0.0, 0.6)	0.4 (0.0, 1.1)	0.0 (0.0, 0.0)
West Africa	Benin	35.9 (31.4, 38.0)	18.7 (10.1, 19.4)	3.2 (1.4, 5.8)	12.4 (5.2, 16.6)	1.7 (0.2, 2.6)	1.7 (0.9, 2.3)	4.2 (2.1, 5.2)	1.5 (0.4, 2.4)	3.2 (1.1, 4.4)	0.9 (0.5, 1.1)	0.0 (0.0, 6.8)	0.1 (0.1, 0.1)
	Burkina Faso	33.3 (23.7, 38.0)	9.6 (7.3, 10.8)	5.8 (4.3, 7.0)	4.2 (1.5, 5.7)	4.3 (2.8, 5.5)	0.8 (0.6, 1.0)	3.7 (1.8, 5.1)	2.7 (1.8, 3.3)	1.1 (0.4, 1.7)	0.5 (0.0, 0.7)	0.6 (0.0, 1.3)	0.0 (0.0, 0.0)
	Côte d'Ivoire	9.3 (7.4, 10.4)	10.7 (7.8, 12.2)	1.1 (0.7, 1.5)	3.3 (0.3, 5.7)	2.9 (2.3, 3.4)	3.6 (2.3, 4.5)	2.2 (1.4, 3.0)	0.6 (0.3, 0.8)	1.2 (0.1, 2.3)	0.6 (0.0, 1.8)	2.2 (0.0, 4.6)	0.0 (0.0, 0.0)
	Ghana	35.6 (27.2, 39.0)	8.2 (3.6, 10.0)	6.6 (5.5, 7.6)	4.5 (1.2, 7.2)	2.6 (1.6, 3.4)	2.1 (1.4, 2.6)	5.0 (1.7, 6.9)	0.9 (0.6, 1.1)	2.5 (0.7, 3.8)	1.3 (0.6, 1.9)	2.1 (0.0, 3.5)	0.0 (0.0, 0.0)
	Nigeria	52.7 (45.7, 58.8)	9.6 (4.8, 12.7)	4.1 (1.6, 4.9)	5.5 (1.8, 7.2)	4.9 (1.6, 6.5)	6.6 (2.9, 7.9)	3.4 (1.5, 5.5)	4.0 (2.2, 5.1)	0.1 (0.0, 0.2)	2.3 (0.5, 3.2)	0.2 (0.0, 0.5)	0.1 (0.1, 0.1)
East and Southern Africa	Ethiopia	12.9 (10.6, 15.1)	17.8 (14.0, 20.4)	6.5 (2.6, 7.7)	1.8 (0.0, 2.7)	4.6 (2.9, 5.7)	6.9 (4.2, 8.5)	5.2 (2.7, 7.4)	3.9 (2.5, 4.9)	2.6 (0.0, 3.6)	0.0 (0.0, 0.0)	1.7 (0.0, 3.2)	0.7 (0.6, 0.7)
	Madagascar	28.4 (22.4, 30.8)	14.7 (9.8, 17.4)	11.5 (4.6, 14.1)	8.3 (0.0, 13.3)	4.6 (1.8, 5.9)	6.2 (2.0, 7.6)	3.2 (1.3, 4.6)	1.3 (0.7, 1.7)	3.3 (1.1, 4.6)	1.3 (0.0, 1.7)	1.4 (0.0, 3.1)	0.0 (0.0, 0.0)
	Mauritius	11.5 (9.1, 12.9)	1.9 (1.5, 2.2)	18.0 (11.9, 20.0)	12.7 (1.2, 18.4)	6.3 (4.5, 7.8)	1.5 (1.0, 1.9)	0.2 (0.1, 0.3)	3.1 (2.1, 3.8)	2.0 (0.4, 3.1)	2.6 (0.0, 3.8)	0.9 (0.0, 2.4)	0.0 (0.0, 0.0)
	Uganda	26.5 (20.3, 30.5)	6.1 (4.8, 7.1)	4.5 (3.0, 5.6)	5.8 (0.1, 9.5)	5.6 (4.5, 6.8)	5.5 (4.1, 6.9)	9.2 (5.2, 11.9)	1.7 (0.8, 2.3)	0.9 (0.1, 1.5)	0.4 (0.0, 1.1)	2.6 (0.0, 5.0)	1.7 (1.6, 1.8)
	Zambia	46.2 (35.3, 48.7)	13.5 (10.3, 16.6)	10.0 (3.3, 19.9)	20.4 (15.5, 28.6)	3.5 (0.0, 8.7)	7.1 (4.2, 9.4)	13.2 (5.9, 15.2)	1.3 (0.1, 2.5)	2.7 (0.0, 4.9)	2.3 (0.5, 2.7)	0.0 (0.0, 6.0)	1.0 (0.9, 1.0)
	Zimbabwe	29.8 (22.3, 32.8)	9.2 (6.0, 10.9)	10.7 (8.7, 12.4)	4.0 (0.0, 6.8)	4.7 (3.1, 6.1)	3.1 (2.0, 3.9)	2.2 (1.3, 3.1)	0.6 (0.3, 0.8)	1.8 (0.5, 2.4)	4.6 (1.9, 5.6)	0.4 (0.0, 0.9)	0.0 (0.0, 0.0)
South Asia	India #1	24.3 (20.0, 26.8)	14.4 (5.8, 18.0)	9.7 (2.9, 11.9)	24.5 (5.6, 34.2)	4.4 (0.3, 5.8)	1.7 (0.7, 2.2)	0.6 (0.3, 0.9)	1.7 (0.8, 2.3)	2.2 (0.7, 3.3)	2.7 (0.5, 3.3)	1.0 (0.0, 3.8)	0.1 (0.1, 0.2)
	India #2	34.0 (28.1, 36.9)	7.9 (3.5, 10.7)	16.1 (5.4, 19.5)	14.5 (4.4, 22.0)	5.1 (0.5, 7.0)	1.7 (0.7, 2.2)	0.5 (0.2, 0.8)	1.3 (0.4, 2.0)	6.3 (1.3, 9.0)	1.4 (0.4, 1.7)	0.0 (0.0, 0.2)	0.0 (0.0, 0.0)
	India #3	13.5 (10.1, 15.1)	15.9 (5.8, 21.1)	7.4 (2.1, 9.5)	15.4 (2.7, 24.2)	7.7 (1.7, 10.3)	2.4 (0.8, 3.1)	3.3 (1.1, 5.2)	6.8 (2.8, 9.0)	2.3 (0.1, 3.7)	0.0 (0.0, 0.1)	1.1 (0.0, 5.0)	7.0 (6.1, 7.4)
	India #4	7.2 (5.3, 8.3)	10.5 (4.5, 14.8)	7.0 (2.1, 9.2)	25.7 (13.4, 35.8)	12.9 (1.6, 18.6)	2.7 (0.8, 3.6)	0.0 (0.0, 0.0)	15.5 (3.4, 21.6)	1.2 (0.2, 2.0)	0.7 (0.1, 1.0)	1.1 (0.0, 5.1)	0.0 (0.0, 0.0)
	India #5*	---	---	---	---	---	---	---	---	---	---	---	---
	Pakistan #1*	---	---	---	---	---	---	---	---	---	---	---	---
	Pakistan #2*	---	---	---	---	---	---	---	---	---	---	---	---
Southeast Asia and Oceania	Fiji	14.9 (11.9, 16.4)	18.4 (14.6, 20.0)	14.4 (5.4, 16.7)	8.0 (0.1, 10.6)	5.3 (3.1, 6.7)	4.4 (3.1, 5.2)	0.9 (0.6, 1.4)	2.2 (1.3, 2.7)	2.2 (0.4, 3.2)	0.0 (0.0, 0.0)	2.5 (0.0, 4.9)	0.0 (0.0, 0.0)
	Indonesia	26.9 (19.4, 30.0)	9.1 (7.4, 10.3)	7.8 (6.4, 8.5)	4.9 (2.0, 6.4)	1.4 (0.9, 1.6)	1.0 (0.7, 1.2)	0.2 (0.1, 0.3)	2.7 (2.1, 3.3)	3.4 (1.8, 4.6)	0.5 (0.0, 0.9)	0.0 (0.0, 0.0)	0.6 (0.5, 0.6)
	Lao PDR	40.5 (32.2, 44.4)	4.6 (3.5, 5.1)	12.0 (9.8, 13.1)	1.5 (0.5, 1.8)	3.8 (2.6, 4.6)	1.0 (0.6, 1.2)	1.2 (0.6, 1.8)	0.4 (0.2, 0.5)	2.0 (0.9, 2.9)	2.4 (0.4, 4.0)	0.2 (0.0, 0.4)	0.0 (0.0, 0.0)
	Myanmar	59.9 (53.9, 63.4)	3.7 (2.9, 5.4)	5.8 (3.6, 8.2)	15.4 (0.1, 19.2)	3.1 (0.0, 4.8)	2.6 (1.1, 3.9)	1.0 (0.6, 1.2)	3.9 (1.0, 5.6)	0.7 (0.0, 2.3)	0.3 (0.0, 0.4)	0.0 (0.0, 2.6)	1.2 (0.9, 1.4)
	Philippines*	---	---	---	---	---	---	---	---	---	---	---	---
	Viet Nam	21.8 (16.2, 24.4)	1.3 (1.0, 1.4)	19.4 (15.9, 21.6)	1.3 (0.4, 1.7)	3.7 (2.9, 4.4)	1.0 (0.7, 1.3)	0.1 (0.1, 0.1)	0.9 (0.5, 1.2)	3.0 (1.0, 4.0)	0.2 (0.0, 0.3)	0.0 (0.0, 0.0)	0.0 (0.0, 0.0)
East Asia	China #1	18.5 (13.9, 20.9)	0.0 (0.0, 0.0)	5.6 (4.6, 6.7)	3.1 (1.6, 4.3)	1.0 (0.8, 1.1)	0.0 (0.0, 0.0)	0.0 (0.0, 0.0)	1.3 (0.8, 1.7)	0.4 (0.1, 0.5)	0.9 (0.0, 1.3)	0.0 (0.0, 0.1)	0.0 (0.0, 0.0)
	China #2	21.9 (16.5, 23.8)	0.0 (0.0, 0.0)	8.8 (7.2, 9.7)	1.2 (0.6, 1.6)	1.9 (1.4, 2.1)	0.2 (0.1, 0.2)	0.0 (0.0, 0.0)	1.4 (0.8, 1.7)	1.0 (0.4, 1.5)	1.6 (0.4, 2.5)	0.0 (0.0, 0.0)	0.0 (0.0, 0.0)
	China #3	43.4 (31.9, 48.1)	0.0 (0.0, 0.0)	23.4 (19.1, 25.8)	2.4 (1.2, 3.5)	1.1 (0.8, 1.4)	0.0 (0.0, 0.0)	0.0 (0.0, 0.0)	1.9 (1.1, 2.4)	0.8 (0.2, 1.1)	1.0 (0.1, 1.4)	0.3 (0.0, 0.7)	0.0 (0.0, 0.0)

Attributable fractions are expressed as a percent. Lao PDR=Lao People's Democratic Republic. tEPEC=typical enteropathogenic *E. coli*. ETEC=enterotoxigenic *E. coli*.
*Site did not participate in GPDS during this two-year time period

Table F. All-cause and pathogen-specific attributable incidence per 1000 child-years with 95% confidence intervals of diarrhea hospitalizations in children aged <5 years both overall and by geographic grouping in countries participating in Global Pediatric Diarrhea Surveillance, by two-year time periods during 2017-2022 (Underlying data for Figure 6).

	All-cause	Rotavirus	*Shigella*	Norovirus	Adenovirus 40/41	Sapovirus	ETEC	*Cryptosporidium*	Astrovirus	*C. jejuni/coli*	*Salmonella*	tEPEC	*V. cholerae*
**2017-2018**													
Overall	11.9 (10.0, 14.3)	4.4 (3.5, 5.6)	1.3 (1.0, 1.6)	0.7 (0.6, 0.9)	0.8 (0.6, 1.1)	0.5 (0.3, 0.7)	0.6 (0.4, 0.8)	0.4 (0.3, 0.6)	0.4 (0.3, 0.5)	0.2 (0.1, 0.2)	0.2 (0.1, 0.3)	0.1 (0.0, 0.1)	0.1 (0.1, 0.1)
Central America	1.8 (1.4, 2.4)	0.4 (0.3, 0.5)	0.3 (0.2, 0.5)	0.3 (0.1, 0.4)	0.2 (0.1, 0.3)	0.1 (0.0, 0.1)	0.0 (0.0, 0.1)	0.0 (0.0, 0.0)	0.1 (0.0, 0.1)	0.1 (0.0, 0.1)	0.0 (0.0, 0.0)	0.0 (0.0, 0.0)	0.0 (0.0, 0.0)
South America	2.2 (1.7, 2.9)	0.3 (0.2, 0.4)	0.3 (0.2, 0.4)	0.5 (0.4, 0.7)	0.1 (0.1, 0.2)	0.1 (0.1, 0.1)	0.1 (0.0, 0.1)	0.0 (0.0, 0.0)	0.0 (0.0, 0.1)	0.1 (0.0, 0.1)	0.0 (0.0, 0.0)	0.0 (0.0, 0.0)	0.0 (0.0, 0.0)
Eastern Europe	1.4 (0.9, 2.1)	0.5 (0.3, 0.7)	0.0 (0.0, 0.0)	0.1 (0.1, 0.2)	0.0 (0.0, 0.0)	0.0 (0.0, 0.1)	0.0 (0.0, 0.0)	0.0 (0.0, 0.0)	0.0 (0.0, 0.0)	0.1 (0.0, 0.1)	0.0 (0.0, 0.0)	0.0 (0.0, 0.0)	0.0 (0.0, 0.0)
Central and Western Asia	1.1 (0.8, 1.4)	0.2 (0.2, 0.3)	0.1 (0.1, 0.2)	0.1 (0.1, 0.1)	0.0 (0.0, 0.1)	0.1 (0.0, 0.1)	0.0 (0.0, 0.1)	0.0 (0.0, 0.1)	0.0 (0.0, 0.1)	0.0 (0.0, 0.0)	0.0 (0.0, 0.0)	0.0 (0.0, 0.0)	0.0 (0.0, 0.0)
West Africa	43.1 (31.8, 57.7)	20.8 (14.6, 29.2)	4.2 (2.4, 6.4)	1.7 (0.9, 2.5)	2.3 (0.9, 3.5)	1.9 (0.6, 2.9)	2.5 (1.0, 3.8)	1.5 (0.7, 2.5)	1.5 (0.7, 2.4)	0.2 (0.1, 0.2)	0.9 (0.2, 1.5)	0.2 (0.1, 0.3)	0.0 (0.0, 0.1)
East and Southern Africa	26.8 (21.6, 33.2)	5.5 (4.5, 6.7)	3.9 (2.8, 5.1)	1.9 (1.3, 2.5)	1.3 (0.8, 1.8)	1.2 (0.9, 1.6)	1.7 (1.2, 2.3)	1.6 (1.1, 2.1)	0.7 (0.5, 1.1)	0.6 (0.2, 0.9)	0.2 (0.1, 0.2)	0.4 (0.1, 0.8)	0.2 (0.2, 0.2)
South Asia	4.9 (3.4, 7.1)	1.2 (0.8, 1.7)	0.6 (0.3, 1.0)	0.5 (0.2, 0.8)	0.8 (0.4, 1.4)	0.3 (0.1, 0.4)	0.1 (0.0, 0.1)	0.1 (0.0, 0.1)	0.2 (0.1, 0.3)	0.2 (0.1, 0.3)	0.1 (0.0, 0.1)	0.1 (0.0, 0.1)	0.1 (0.1, 0.2)
Southeast Asia and Oceania	10.3 (8.2, 13.1)	3.3 (2.6, 4.2)	0.6 (0.4, 0.9)	1.0 (0.8, 1.3)	0.6 (0.3, 0.8)	0.2 (0.1, 0.3)	0.1 (0.1, 0.2)	0.0 (0.0, 0.0)	0.3 (0.2, 0.4)	0.3 (0.2, 0.4)	0.0 (0.0, 0.1)	0.0 (0.0, 0.1)	0.1 (0.0, 0.1)
East Asia	0.7 (0.4, 1.0)	0.2 (0.1, 0.3)	0.0 (0.0, 0.0)	0.1 (0.1, 0.1)	0.0 (0.0, 0.0)	0.0 (0.0, 0.0)	0.0 (0.0, 0.0)	0.0 (0.0, 0.0)	0.0 (0.0, 0.0)	0.0 (0.0, 0.0)	0.0 (0.0, 0.0)	0.0 (0.0, 0.0)	0.0 (0.0, 0.0)
**2019-2020**													
Overall	9.9 (8.3, 11.9)	3.2 (2.3, 4.3)	0.9 (0.7, 1.1)	0.7 (0.5, 0.8)	1.2 (0.6, 1.8)	0.4 (0.2, 0.5)	0.3 (0.3, 0.4)	0.2 (0.1, 0.3)	0.2 (0.1, 0.2)	0.1 (0.1, 0.2)	0.1 (0.0, 0.1)	0.1 (0.0, 0.2)	0.1 (0.1, 0.1)
Central America	1.7 (1.2, 2.3)	0.4 (0.3, 0.5)	0.2 (0.1, 0.3)	0.4 (0.2, 0.5)	0.1 (0.1, 0.2)	0.1 (0.0, 0.1)	0.0 (0.0, 0.0)	0.0 (0.0, 0.0)	0.1 (0.0, 0.1)	0.0 (0.0, 0.0)	0.0 (0.0, 0.0)	0.0 (0.0, 0.0)	0.0 (0.0, 0.0)
South America	1.9 (1.4, 2.4)	0.2 (0.2, 0.3)	0.3 (0.2, 0.4)	0.4 (0.3, 0.5)	0.1 (0.1, 0.2)	0.1 (0.1, 0.1)	0.1 (0.0, 0.1)	0.0 (0.0, 0.0)	0.0 (0.0, 0.0)	0.0 (0.0, 0.1)	0.0 (0.0, 0.0)	0.0 (0.0, 0.0)	0.0 (0.0, 0.0)
Eastern Europe	1.3 (0.8, 1.9)	0.3 (0.2, 0.5)	0.0 (0.0, 0.0)	0.2 (0.1, 0.2)	0.1 (0.0, 0.2)	0.0 (0.0, 0.0)	0.0 (0.0, 0.0)	0.0 (0.0, 0.0)	0.0 (0.0, 0.0)	0.1 (0.0, 0.1)	0.0 (0.0, 0.0)	0.0 (0.0, 0.0)	0.0 (0.0, 0.0)
Central and Western Asia	2.1 (1.5, 2.9)	0.5 (0.3, 0.7)	0.3 (0.2, 0.4)	0.2 (0.2, 0.4)	0.1 (0.1, 0.2)	0.1 (0.1, 0.2)	0.0 (0.0, 0.0)	0.0 (0.0, 0.1)	0.1 (0.0, 0.1)	0.0 (0.0, 0.0)	0.0 (0.0, 0.0)	0.0 (0.0, 0.1)	0.0 (0.0, 0.0)
West Africa	35.4 (26.1, 47.2)	16.2 (10.1, 23.1)	2.0 (1.0, 2.9)	2.2 (1.0, 3.2)	5.0 (1.1, 8.5)	1.3 (0.3, 2.1)	0.8 (0.4, 1.2)	0.6 (0.3, 0.9)	0.4 (0.2, 0.6)	0.3 (0.2, 0.4)	0.2 (0.1, 0.4)	0.3 (0.1, 0.7)	0.0 (0.0, 0.0)
East and Southern Africa	20.0 (16.0, 24.9)	3.5 (2.9, 4.2)	3.1 (2.2, 4.1)	1.5 (0.9, 2.0)	1.4 (0.7, 1.9)	0.9 (0.6, 1.2)	1.0 (0.7, 1.4)	0.7 (0.5, 1.0)	0.6 (0.4, 0.9)	0.4 (0.2, 0.7)	0.0 (0.0, 0.0)	0.2 (0.1, 0.5)	0.0 (0.0, 0.0)
South Asia	4.8 (3.6, 6.3)	0.6 (0.5, 0.8)	0.6 (0.3, 0.9)	0.3 (0.1, 0.5)	0.6 (0.4, 0.9)	0.2 (0.1, 0.3)	0.2 (0.1, 0.4)	0.1 (0.1, 0.1)	0.1 (0.1, 0.1)	0.1 (0.0, 0.2)	0.0 (0.0, 0.1)	0.1 (0.0, 0.2)	0.2 (0.1, 0.2)
Southeast Asia and Oceania	11.3 (8.7, 14.5)	3.7 (2.7, 5.0)	0.3 (0.2, 0.5)	0.9 (0.6, 1.3)	1.1 (0.0, 1.8)	0.2 (0.1, 0.3)	0.1 (0.0, 0.2)	0.0 (0.0, 0.0)	0.1 (0.0, 0.1)	0.2 (0.0, 0.4)	0.1 (0.0, 0.2)	0.0 (0.0, 0.2)	0.0 (0.0, 0.0)
East Asia	0.6 (0.4, 0.9)	0.1 (0.1, 0.2)	0.0 (0.0, 0.0)	0.1 (0.1, 0.1)	0.0 (0.0, 0.0)	0.0 (0.0, 0.0)	0.0 (0.0, 0.0)	0.0 (0.0, 0.0)	0.0 (0.0, 0.0)	0.0 (0.0, 0.0)	0.0 (0.0, 0.0)	0.0 (0.0, 0.0)	0.0 (0.0, 0.0)
**2021-2022**													
Overall	8.8 (7.4, 10.5)	2.3 (1.8, 3.0)	0.9 (0.7, 1.1)	0.6 (0.4, 0.8)	0.6 (0.4, 0.8)	0.2 (0.1, 0.3)	0.2 (0.2, 0.3)	0.2 (0.1, 0.2)	0.1 (0.1, 0.1)	0.1 (0.1, 0.2)	0.1 (0.1, 0.1)	0.1 (0.0, 0.1)	0.1 (0.1, 0.2)
Central America	1.7 (1.3, 2.3)	0.2 (0.2, 0.3)	0.2 (0.1, 0.2)	0.3 (0.1, 0.4)	0.1 (0.0, 0.2)	0.1 (0.0, 0.1)	0.0 (0.0, 0.1)	0.0 (0.0, 0.0)	0.0 (0.0, 0.1)	0.0 (0.0, 0.1)	0.0 (0.0, 0.0)	0.0 (0.0, 0.0)	0.0 (0.0, 0.0)
South America	1.5 (1.1, 1.9)	0.1 (0.1, 0.2)	0.2 (0.1, 0.2)	0.3 (0.2, 0.4)	0.1 (0.1, 0.1)	0.0 (0.0, 0.1)	0.0 (0.0, 0.1)	0.0 (0.0, 0.0)	0.0 (0.0, 0.0)	0.1 (0.0, 0.1)	0.0 (0.0, 0.0)	0.0 (0.0, 0.0)	0.0 (0.0, 0.0)
Eastern Europe	2.0 (1.4, 3.0)	0.5 (0.3, 0.7)	0.0 (0.0, 0.0)	0.3 (0.2, 0.4)	0.1 (0.1, 0.2)	0.0 (0.0, 0.0)	0.0 (0.0, 0.0)	0.0 (0.0, 0.0)	0.0 (0.0, 0.0)	0.1 (0.0, 0.1)	0.0 (0.0, 0.0)	0.0 (0.0, 0.0)	0.0 (0.0, 0.0)
Central and Western Asia	2.0 (1.4, 2.8)	0.3 (0.2, 0.5)	0.2 (0.1, 0.3)	0.2 (0.2, 0.4)	0.1 (0.0, 0.1)	0.1 (0.1, 0.1)	0.0 (0.0, 0.0)	0.0 (0.0, 0.1)	0.0 (0.0, 0.0)	0.0 (0.0, 0.0)	0.0 (0.0, 0.0)	0.0 (0.0, 0.0)	0.0 (0.0, 0.0)
West Africa	29.1 (21.0, 39.6)	10.5 (7.0, 15.0)	1.4 (0.7, 2.0)	2.1 (0.9, 3.1)	2.7 (1.1, 4.3)	0.6 (0.2, 1.0)	0.4 (0.2, 0.6)	0.6 (0.2, 1.0)	0.3 (0.2, 0.5)	0.1 (0.0, 0.1)	0.1 (0.0, 0.1)	0.2 (0.0, 0.4)	0.0 (0.0, 0.0)
East and Southern Africa	18.0 (14.5, 22.5)	3.6 (3.0, 4.4)	3.3 (2.4, 4.5)	1.0 (0.7, 1.4)	0.9 (0.5, 1.3)	0.6 (0.4, 0.8)	1.0 (0.7, 1.2)	0.7 (0.5, 1.0)	0.3 (0.2, 0.4)	0.5 (0.2, 0.7)	0.2 (0.0, 0.4)	0.2 (0.1, 0.4)	0.0 (0.0, 0.0)
South Asia	4.3 (3.2, 5.8)	0.7 (0.5, 0.9)	0.7 (0.3, 1.1)	0.2 (0.1, 0.4)	0.2 (0.1, 0.3)	0.1 (0.0, 0.2)	0.1 (0.1, 0.2)	0.0 (0.0, 0.1)	0.0 (0.0, 0.1)	0.1 (0.0, 0.2)	0.1 (0.0, 0.1)	0.0 (0.0, 0.1)	0.2 (0.1, 0.2)
Southeast Asia and Oceania	10.5 (7.9, 13.9)	2.2 (1.5, 3.0)	0.6 (0.3, 0.9)	1.7 (1.1, 2.4)	0.3 (0.1, 0.5)	0.3 (0.2, 0.5)	0.1 (0.1, 0.2)	0.0 (0.0, 0.0)	0.2 (0.1, 0.3)	0.5 (0.2, 0.8)	0.2 (0.1, 0.4)	0.0 (0.0, 0.1)	0.8 (0.5, 1.2)
East Asia	0.5 (0.3, 0.8)	0.1 (0.1, 0.1)	0.0 (0.0, 0.0)	0.1 (0.0, 0.1)	0.0 (0.0, 0.1)	0.0 (0.0, 0.0)	0.0 (0.0, 0.0)	0.0 (0.0, 0.0)	0.0 (0.0, 0.0)	0.0 (0.0, 0.0)	0.0 (0.0, 0.0)	0.0 (0.0, 0.0)	0.0 (0.0, 0.0)

tEPEC=typical enteropathogenic *E. coli*. ETEC=heat-stable enterotoxigenic *E. coli*

Fig A. Prevalence of pathogens including in the attribution modelling at cycle threshold cutoffs of 35 and 30 in children <5 years hospitalized with diarrhea and enrolled in Global Pediatric Diarrhea Surveillance, by site and two-year time period from 2017-2022.

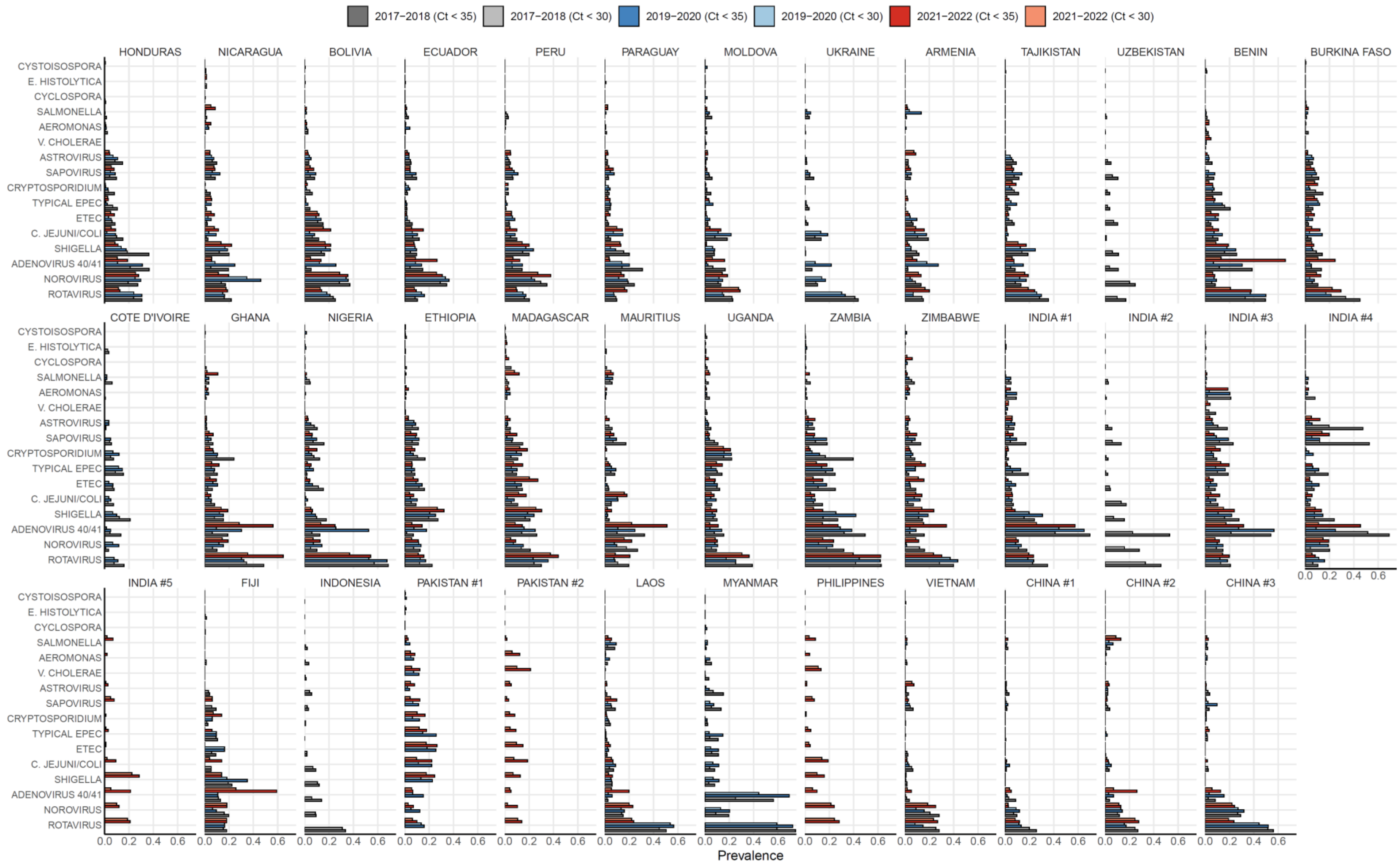

Supplement: Supplementary file 2 [file pgph.0006655.s002.pdf]
