# Supplementary material for: Etiology and incidence of diarrhea requiring hospitalization in children under 5 years of age in 31 low- and middle-income countries: Findings from the Global Pediatric Diarrhea Surveillance network, 2017–2022
Source: PLOS Glob Public Health. 2026 Oct 27;6(10):e0006655. doi: 10.1371/journal.pgph.0006655 (PMC13712721; doi:10.1371/journal.pgph.0006655)
Supplement: Supplementary file 3 [file pgph.0006655.s004.pdf]

Editor comments

Figure 1: please (a) provide a direct link to the base layer of the map (i.e., the country or region border shape) and ensure this is also included in the figure legend; and (b) provide a link to the terms of use / license information for the base layer image or shapefile. We cannot publish proprietary or copyrighted maps (e.g. Google Maps, Mapquest) and the terms of use for your map base layer must be compatible with our CC-BY 4.0 license.

Response: The base map boundaries in Figure 1 were generated using the open-source R package rworldmap, which derives its country border shapefiles from Natural Earth.

1. **Base layer source:** Natural Earth 1:50m Admin 0 Country Boundaries (https://www.naturalearthdata.com/downloads/50m-cultural-vectors/50m-admin-0-countries-2/).
2. **Terms of use / license:** Natural Earth data are in the public domain and free for all uses (https://www.naturalearthdata.com/about/terms-of-use/)

We have added the link to the base layer to the Figure 1 legend.

Reviewer #1

1. Review grammar: a few minor errors e.g. through instead of though (line 142); Sample were (line 157)

Response: We thank the reviewer for this observation. The grammar has been reviewed and these minor errors were corrected.

2. Methods: Need to acknowledge limitations of use of other modelling studies in your analysis i.e. using models developed from qPCR reanalyses of the GEMS case-control study and the MAL-ED birth cohort study, and use of GBD estimates. These studies/models themselves have certain assumptions and potential biases e.g. GEMS/MAL-ED were not global studies and case-control studies are prone to bias especially with regards to control selection.

Response: We agree with the reviewer that this is an important limitation. We have previously addressed this limitation in detail [Cohen et al: https://gh.bmj.com/content/7/9/e009548], but we agree that it should be repeated here. Thus, we have added the following sentences to the Discussion:

> *As GPDS estimates incorporate data from GBD (17), and GPDS does not enroll non-diarrheal controls but rather relies on attribution models developed in previous studies (14–16), the limitations of GBD and the previous studies inherently apply to GPDS as well. We previously addressed the limitations of the attribution models in detail (11). However, it is reassuring that in this analysis, the top four attributable pathogens were also the four pathogens with the highest prevalence. Therefore, while the attribution models make the estimated absolute contribution of each pathogen more conservative, they do not change the pathogen hierarchy.*

3. Methods, Line 182-3: Not clear why analyses were run using GPDS surveillance data available as of 16 January 2026, yet you only provide results from 2017-2022. Were data not available for subsequent years? It would have been good to have additional post-COVID-19 years included in the analyses to observe trends in subsequent years.

Response: At the time of these analyses, GPDS data for subsequent years were not yet available. GPDS testing is done retrospectively following the completion of each surveillance year, and the data is analyzed in two-year increments. At the time of these analyses, the GPDS dataset for 2023-2024 was not yet complete, validated, and finalized. Future analyses of GPDS data will be able to explore more post-COVID-19 trends, as the reviewer suggests.

4. Discussion (line 326): In additional to good uptake in the Americas, the efficacy of rotavirus vaccines is also higher in low mortality countries compared to high mortality countries. This should be mentioned in the discussion.

Response: We thank the reviewer for this observation. Accordingly, we have revised this sentence and added reference 20:

> *GPDS data have quantified a consistently high burden of norovirus in the Americas, the leading cause of diarrhea hospitalizations in a region that introduced rotavirus vaccines over a decade ago with good uptake* ***and high efficacy of vaccines in clinical trials*** **(20)***; this finding is consistent with previous reports (21–23).*

5. Line 339-341: it's not clear why banking and retrospective testing of stool samples helped sustain surveillance activities during the pandemic. Unless you are implying that testing of samples was able to continue despite the pandemic? Suggest revising the sentence for clarity.

Response: Thank you to the reviewer for highlighting the need for improved clarity. We have revised this sentence which now reads as follows:

> *Although many efforts were made to sustain GPDS surveillance during the pandemic, and GPDS has a relatively forgiving study design of banking and retrospective testing of stool samples* ***rather than requiring real-time laboratory testing*** *(10), a number of sites experienced surveillance interruptions.*

<u>Reviewer #2</u>

1. Although it is internally correct, the use of different denominators "...enrolled 70,750 children aged <5 years hospitalized with diarrhea, of which 16,458 (23.3%)" in the abstract and "…..68,302 (96.5%) had a stool sample collected and were eligible for qPCR testing; of these, 16,458 (24.1%) were selected" might be slightly confusing readers. I suggest either using one denominator consistently or explicitly labelling which denominator each percentage refers to.

Response: We thank the reviewer for noticing this issue. As suggested, we have chosen to use one denominator consistently for the Abstract, Results, and Table 1: the total number of enrolled cases. We have revised this sentence of the Results accordingly:

> *Of the 70,750 enrolled cases, 68,302 (96.5%) had a stool sample collected and ~~were eligible for qPCR testing; of these,~~ 16,458 (***23.3%***) were* ***randomly*** *selected and tested: 5,670 from 2017-2018, 5,509 from 2019-2020, and 5,279 from 2021-2022 (Table 1).*

2. In discussion 322… "Rotavirus vaccine product disruptions" is listed alongside two other possible factors, both of which are otherwise discussed or documented in the manuscript. The vaccine product disruption factor is not mentioned anywhere else in the paper, and no supporting reference is given. Since this is a

specific factual claim about a real-world supply event rather than a general possibility, I suggest either adding a citation if such disruptions did occur in specific GPDS countries during this period or removing it from the list if it was included speculatively without a known basis

Response: Thank you for this suggestion. We have added 2 references with details about the rotavirus vaccine product disruptions, which did occur in some GPDS countries during this period. We've also re-ordered this sentence to make the product disruptions the third potential factor listed instead of the first. These sentences now read as follows:

> *Among the subset of countries with rotavirus vaccine, 5 of 9 regions experienced an increase in rotavirus AF in 2021-2022 as compared to 2019-2020. This may have been due to a variety of factors, including ~~rotavirus vaccine product disruptions,~~ COVID-19 pandemic impacts on disease transmission and surveillance, ~~and~~ some changes in GPDS site participation over time***, and rotavirus vaccine product disruptions (18,19).***
