# Supplementary material for: Etiology and incidence of diarrhea requiring hospitalization in children under 5 years of age in 31 low- and middle-income countries: Findings from the Global Pediatric Diarrhea Surveillance network, 2017–2022
Source: PLOS Glob Public Health. 2026 Oct 27;6(10):e0006655. doi: 10.1371/journal.pgph.0006655 (PMC13712721; doi:10.1371/journal.pgph.0006655)
Supplement: Peer review history

# Peer review: Decision Letter 0

**DOI:** 10.1371/journal.pgph.0006655.r001

**Authors:** Ioana D. Olaru

16 Sep 2026

PGPH-D-26-02007

Etiology and incidence of diarrhea requiring hospitalization in children under 5 years of age in 31 low- and middle-income countries: findings from the Global Pediatric Diarrhea Surveillance network, 2017–2022

PLOS Global Public Health

Dear Dr. Soeters,

Thank you for submitting your manuscript to PLOS Global Public Health. After careful consideration, we feel that it has merit but does not fully meet PLOS Global Public Health’s publication criteria as it currently stands. Therefore, we invite you to submit a revised version of the manuscript that addresses the points raised during the review process.

Please submit your revised manuscript by Oct 16 2026 11:59PM. If you will need more time than this to complete your revisions, please reply to this message or contact the journal office at globalpubhealth@plos.org. When you're ready to submit your revision, log on to https://www.editorialmanager.com/pgph/ and select the 'Submissions Needing Revision' folder to locate your manuscript file.

Please include the following items when submitting your revised manuscript:

- A letter that responds to each point raised by the editor and reviewer(s). You should upload this letter as a separate file labeled 'Response to Reviewers'.
- A marked-up copy of your manuscript that highlights changes made to the original version. You should upload this as a separate file labeled 'Revised Manuscript with Track Changes'.
- An unmarked version of your revised paper without tracked changes. You should upload this as a separate file labeled 'Manuscript'.

Guidelines for resubmitting your figure files are available below the reviewer comments at the end of this letter.

As the corresponding author, your ORCID iD is verified in the submission system and will appear in the published article. PLOS supports the use of ORCID, and we encourage all coauthors to register for an ORCID iD and use it as well. Please encourage your coauthors to verify their ORCID iD within the submission system before final acceptance, as unverified ORCID iDs will not appear in the published article. *Only*  the individual author can complete the verification step; PLOS staff *cannot*  verify ORCID iDs on behalf of authors.

We look forward to receiving your revised manuscript.

Kind regards,

Ioana D. Olaru

Academic Editor

PLOS Global Public Health

**Journal Requirements:**

1. For studies involving third-party data, we encourage authors to share any data specific to their analyses that they can legally distribute. PLOS recognizes, however, that authors may be using third-party data they do not have the rights to share. When third-party data cannot be publicly shared, authors must provide all information necessary for interested researchers to apply to gain access to the data.

https://journals.plos.org/globalpublichealth/s/data-availability#loc-acceptable-data-access-restrictions

For any third-party data that the authors cannot legally distribute, they should include the following information in their Data Availability Statement upon submission:

1) A description of the data set and the third-party source

2) If applicable, verification of permission to use the data set

3) Confirmation of whether the authors received any special privileges in accessing the data that other researchers would not have

4) All necessary contact information others would need to apply to gain access to the data

2. Some material included in your submission may be copyrighted. According to PLOS’s copyright policy, authors who use figures or other material (e.g., graphics, clipart, maps) from another author or copyright holder must demonstrate or obtain permission to publish this material under the Creative Commons Attribution 4.0 International (CC BY 4.0) License used by PLOS journals. Please closely review the details of PLOS’s copyright requirements here: PLOS Licenses and Copyright. If you need to request permissions from a copyright holder, you may use PLOS's Copyright Content Permission form.

Please respond directly to this email or email the journal office and provide any known details concerning your material's license terms and permissions required for reuse, even if you have not yet obtained copyright permissions or are unsure of your material's copyright compatibility.

Potential Copyright Issues:

a. Figure 1: please (a) provide a direct link to the base layer of the map (i.e., the country or region border shape) and ensure this is also included in the figure legend; and (b) provide a link to the terms of use / license information for the base layer image or shapefile. We cannot publish proprietary or copyrighted maps (e.g. Google Maps, Mapquest) and the terms of use for your map base layer must be compatible with our CC-BY 4.0 license.

Note: if you created the map in a software program like R or ArcGIS, please locate and indicate the source of the basemap shapefile onto which data has been plotted.

If your map was obtained from a copyrighted source please amend the figure so that the base map used is from an openly available source. Alternatively, please provide explicit written permission from the copyright holder granting you the right to publish the material under our CC-BY 4.0 license.

Please note that the following CC BY licenses are compatible with PLOS license: CC BY 4.0, CC BY 2.0 and CC BY 3.0, meanwhile such licenses as CC BY-ND 3.0 and others are not compatible due to additional restrictions.

If you are unsure whether you can use a map or not, please do reach out and we will be able to help you. The following websites are good examples of where you can source open access or public domain maps:

* U.S. Geological Survey (USGS) - All maps are in the public domain. (http://www.usgs.gov)

* PlaniGlobe - All maps are published under a Creative Commons license so please cite “PlaniGlobe, http://www.planiglobe.com, CC BY 2.0” in the image credit after the caption. (http://www.planiglobe.com/?lang=enl)

* Natural Earth - All maps are public domain. (http://www.naturalearthdata.com/about/terms-of-use/)

3. Please ensure that your Ethics Statement is available in its entirety at the beginning of your Methods section, under a subheading 'Ethics Statement'.

4. Please provide separate figure files in .tif or .eps format.

For more information about figure files please see our guidelines:  https://journals.plos.org/globalpublichealth/s/figures

https://journals.plos.org/globalpublichealth/s/figures#loc-file-requirements

If the reviewer comments include a recommendation to cite specific previously published works, please review and evaluate these publications to determine whether they are relevant and should be cited. There is no requirement to cite these works unless the editor has indicated otherwise.

Please review your reference list to ensure that it is complete and correct. If you have cited papers that have been retracted, please include the rationale for doing so in the manuscript text, or remove these references and replace them with relevant current references. Any changes to the reference list should be mentioned in the rebuttal letter that accompanies your revised manuscript. If you need to cite a retracted article, indicate the article’s retracted status in the References list and also include a citation and full reference for the retraction notice.

**Additional Editor Comments (if provided):**

[Note: HTML markup is below. Please do not edit.]

Reviewers' comments:

Reviewer's Responses to Questions

**Comments to the Author**

1. Does this manuscript meet PLOS Global Public Health’s publication criteria?

Reviewer #1: Yes

Reviewer #2: Yes

**********

2. Has the statistical analysis been performed appropriately and rigorously?

Reviewer #1: Yes

Reviewer #2: Yes

**********

3. Have the authors made all data underlying the findings in their manuscript fully available (please refer to the Data Availability Statement at the start of the manuscript PDF file)?

The PLOS Data policy

Reviewer #1: Yes

Reviewer #2: Yes

**********

4. Is the manuscript presented in an intelligible fashion and written in standard English?

Reviewer #1: Yes

Reviewer #2: Yes

**********

**Reviewer #1:**  Systematically collected and analyzed surveillance data on the etiologies of diarrhea in hospitalized children has been limited, particularly in LMICs where the burden is highest. This study uses data from the Global Pediatric Diarrhea Surveillance (GPDS) network for the period 2017-2022 to estimate pathogen-specific attributable fractions (AFs) and incidence of diarrheal hospitalizations at the global, regional, and country levels. Thirty-eight sentinel surveillance sites in 31 countries across all 6 WHO regions contributed data and samples were tested using custom-designed TAC qPCR cards for 16 enteric pathogens that have been associated with pediatric diarrhea. This is a well-written study showing that despite the widespread use of rotavirus vaccines, rotavirus remained the leading cause of severe diarrhea among children aged <5 years in LMICs globally. Shigella, norovirus, and enteric adenoviruses were also associated with a substantial burden of disease. There were notable differences in the distribution of diarrheal etiologies between regions and across time periods. These data are needed to help prioritize and evaluate the use of existing and upcoming vaccines and interventions.

Minor comments:

- Review grammar: a few minor errors e.g. through instead of though (line 142); Sample were (line 157)

- Methods: Need to acknowledge limitations of use of other modelling studies in your analysis i.e. using models developed from qPCR reanalyses of the GEMS case-control study and the MAL-ED birth cohort study, and use of GBD estimates. These studies/models themselves have certain assumptions and potential biases e.g. GEMS/MAL-ED were not global studies and case-control studies are prone to bias especially with regards to control selection.

- Methods, Line 182-3: Not clear why analyses were run using GPDS surveillance data available as of 16 January 2026, yet you only provide results from 2017-2022. Were data not available for subsequent years? It would have been good to have additional post-COVID-19 years included in the analyses to observe trends in subsequent years.

- Discussion (line 326): In additional to good uptake in the Americas, the efficacy of rotavirus vaccines is also higher in low mortality countries compared to high mortality countries. This should be mentioned in the discussion.

- Line 339-341: it’s not clear why banking and retrospective testing of stool samples helped sustain surveillance activities during the pandemic. Unless you are implying that testing of samples was able to continue despite the pandemic? Suggest revising the sentence for clarity.

**Reviewer #2:**  The study reports an important topic, a very interesting manuscript to read, and is very well prepared. Well done.

Few observations

1. Although it is internally correct, the use of different denominators “...enrolled 70,750 children aged <5 years hospitalized with diarrhea, of which 16,458 (23.3%)” in the abstract and “…..68,302 (96.5%) had a stool sample collected and were eligible for qPCR testing; of these, 16,458 (24.1%) were selected” might be slightly confusing readers. I suggest either using one denominator consistently or explicitly labelling which denominator each percentage refers to.

2. In discussion 322… "Rotavirus vaccine product disruptions" is listed alongside two other possible factors, both of which are otherwise discussed or documented in the manuscript. The vaccine product disruption factor is not mentioned anywhere else in the paper, and no supporting reference is given. Since this is a specific factual claim about a real-world supply event rather than a general possibility, I suggest either adding a citation if such disruptions did occur in specific GPDS countries during this period or removing it from the list if it was included speculatively without a known basis

**********

what does this mean?). If published, this will include your full peer review and any attached files.

**Do you want your identity to be public for this peer review?**  If you choose “no”, your identity will remain anonymous but your review may still be made public.

For information about this choice, including consent withdrawal, please see our Privacy Policy

Reviewer #1: No

Reviewer #2: No

**********

[NOTE: If reviewer comments were submitted as an attachment file, they will be attached to this email and accessible via the submission site. Please log into your account, locate the manuscript record, and check for the action link "View Attachments". If this link does not appear, there are no attachment files.]

**Figure Resubmissions:**

# Author comment: Author response to Decision Letter 1

**DOI:** 10.1371/journal.pgph.0006655.r002

17 Sep 2026

Attachment Submitted filename: Response to Reviewers.pdf

# Editor report: Decision Letter 1

**DOI:** 10.1371/journal.pgph.0006655.r003

**Authors:** Ioana D. Olaru

22 Sep 2026

Etiology and incidence of diarrhea requiring hospitalization in children under 5 years of age in 31 low- and middle-income countries: findings from the Global Pediatric Diarrhea Surveillance network, 2017–2022

PGPH-D-26-02007R1

Dear Dr. Soeters,

We are pleased to inform you that your manuscript 'Etiology and incidence of diarrhea requiring hospitalization in children under 5 years of age in 31 low- and middle-income countries: findings from the Global Pediatric Diarrhea Surveillance network, 2017–2022' has been provisionally accepted for publication in PLOS Global Public Health.

Before your manuscript can be formally accepted you will need to complete some formatting changes, which you will receive in a follow up email. A member of our team will be in touch with a set of requests.

Please note that your manuscript will not be scheduled for publication until you have made the required changes, so a swift response is appreciated.

IMPORTANT: The editorial review process is now complete. PLOS will only permit corrections to spelling, formatting or significant scientific errors from this point onwards. Requests for major changes, or any which affect the scientific understanding of your work, will cause delays to the publication date of your manuscript.

If your institution or institutions have a press office, please notify them about your upcoming paper to help maximize its impact. If they'll be preparing press materials, please inform our press team as soon as possible -- no later than 48 hours after receiving the formal acceptance. Your manuscript will remain under strict press embargo until 2 pm Eastern Time on the date of publication. For more information, please contact globalpubhealth@plos.org.

Thank you again for supporting Open Access publishing; we are looking forward to publishing your work in PLOS Global Public Health.

Best regards,

Ioana D. Olaru

Academic Editor

PLOS Global Public Health

***********************************************************
